# The Protein L-Isoaspartyl (D-Aspartyl) Methyltransferase Regulates Glial-to-Mesenchymal Transition and Migration Induced by TGF-β1 in Human U-87 MG Glioma Cells

**DOI:** 10.3390/ijms23105698

**Published:** 2022-05-19

**Authors:** Fatima Belkourchia, Richard R. Desrosiers

**Affiliations:** Département de Chimie, Université du Québec à Montréal, C.P. 8888, Succursale Centre-Ville, Montréal, QC H3C 3P8, Canada; belkourchia.fatima@courrier.uqam.ca

**Keywords:** protein L-isoaspartyl (D-aspartyl) methyltransferase, PIMT, U-87 glioma cells, slug, snail

## Abstract

The enzyme PIMT methylates abnormal aspartyl residues in proteins. U-87 MG cells are commonly used to study the most frequent brain tumor, glioblastoma. Previously, we reported that PIMT isoform I possessed oncogenic features when overexpressed in U-87 MG and U-251 MG glioma cells. Higher levels of wild-type PIMT stimulated migration and invasion in both glioma cell lines. Conversely, PIMT silencing reduced these migratory abilities of both cell lines. These results indicate that PIMT could play a critical role in glioblastoma growth. Here, we investigated for the first time, molecular mechanisms involving PIMT in the regulation of epithelial to mesenchymal transition (EMT) upon TGF-β1 treatments. Gene array analyses indicated that EMT genes but not PIMT gene were regulated in U-87 MG cells treated with TGF-β1. Importantly, PIMT silencing by siRNA inhibited in vitro migration in U-87 MG cells induced by TGF-β1. In contrast, overexpressed wild-type PIMT and TGF-β1 had additive effects on cell migration. When PIMT was inhibited by siRNA, this prevented Slug induction by TGF-β1, while Snail stimulation by TGF-β1 was increased. Indeed, overexpression of wild-type PIMT led to the opposite effects on Slug and Snail expression dependent on TGF-β1. These data highlighted the importance of PIMT in the EMT response dependent on TGF-β1 in U-87 MG glioma cells by an antagonist regulation in the expression of transcription factors Slug and Snail, which are critical players in EMT.

## 1. Introduction

Adult-type diffuse gliomas that include glioblastoma (GBM) are the most malignant, aggressive, and invasive primary brain tumors characterized with high vascularization. GBM therapy efficacies including surgery, radiotherapy, and chemotherapy with temozolomide are limited, since the median survival of patients is less than 15-months [1]. A typical feature of cancers is genome dysregulation [2] and, consequently, that of signaling pathways. Indeed, signaling pathways are also dysregulated in GBM [1]. Thus, further investigations are necessary to identify dysregulated molecular mechanisms that could serve as potential new targets in GBM treatments.

The protein L-isoaspartyl (D-aspartyl) methyltransferase (PIMT) is a ubiquitous repair enzyme and is most abundant in brain [3]. PIMT recognizes and methylates proteins containing atypical L-isoaspartyl residues and, to a lesser degree, D-aspartyl residues [4]. Few studies have demonstrated PIMT molecular mechanisms in tumor progression and particularly in glioblastoma growth. Previously, our laboratory reported that PIMT possesses pro-angiogenic properties [5]. In fact, PIMT downregulated by siRNA in human umbilical vein endothelial cells (HUVECs) decreased cell migration and formation of capillary-like structures in vitro that was stimulated by vascular endothelial growth factor (VEGF), while HUVEC cells that overexpressed wild-type PIMT promoted these processes in the presence of VEGF [5]. Interestingly, PIMT amounts increased when endothelial cells, such as HUVECs and the bovine aortic endothelial cells, and two human cancer cell lines such as the renal carcinoma cells Caki-1 and the astrocytoma cells U-87 MG, were detached from extracellular matrix (ECM). Conversely, PIMT levels decreased upon re-adhesion to different proteins of ECM [6]. Another study reported similar effects in MDA-MB-231 human breast cancer cells [7]. On the other hand, PIMT acted as an anti-apoptotic protein in different cell types including in brain cells [8]. For instance, PIMT protected cancer cells against apoptosis by methylation of asparaginyl residues 29 and 30 of p53, thus increasing its interaction with HDM2 [9]. Recently, we demonstrated oncogenic properties of PIMT isoform I in U-87 MG and U-251 MG glioma cells [10]. Our results showed a key role for PIMT levels and mainly of its catalytic activity in migration and invasion in both glioma cell lines. In addition, we found that PIMT overexpression and its silencing altered F-actin polymerization and microtubules formation in U-87 MG cells [10]. Together, these results indicate that PIMT could play a critical role in tumor growth and notably in GBM. However, the molecular mechanisms involving PIMT in GBM growth remain to be investigated. 

Alternative splicing of the single human *PCMT* gene leads to two major PIMT isoforms that differ only at the C-terminus [11]. While PIMT isoform I ends with the -RWK sequence, isoform II ends with a -RDEL sequence [11]. Interestingly, our RT-PCR analysis showed that type II PIMT mRNA levels decreased (by about 27%) as observed at PIMT protein levels, while in contrast, the amount of type I PIMT mRNA was higher by 2.3-fold in GBM tissues [12]. Similarly, a higher expression of mRNA PCMT1 was observed and could be used as a biomarker to evaluate patient prognosis in head and neck squamous cell carcinoma [13]. When MDA-MB-231 breast cancer cells were detached from ECM, the epithelial to mesenchymal transition (EMT) response was induced and several EMT genes were upregulated, such as integrin αv, TGF-β1, Snail, Slug, and MMP-2 [7]. Importantly, PIMT inhibition by siRNA prevented the induction of EMT markers, suggesting that PIMT was an essential step to the EMT transformation of these cells when they were detached from the ECM [7]. The EMT is involved in different biological processes such as embryonic development (EMT type I), wound healing (EMT type II) and in cancer cells (EMT type III) that gives, in this latter case, the capacity to these cells to migrate, invade and form metastases [14]. The prognosis of a patient with metastases is a crucial factor for survival, and metastases cause a large percentage of patients to die [15]. Indeed, most studies about EMT mechanisms have been performed in epithelial cancer cells [15] while fewer studies have been done on nervous system cancers. Different EMT markers and specific EMT molecular mechanisms seems to be involved in GBM growth. For example, E-cadherin protein is rarely expressed in gliomas [16]. Thus, it is important to determine which molecular mechanisms in EMT allow glioma cells to migrate, invade, and resist apoptosis, and thus to form metastases. 

To better understand the molecular mechanisms in EMT responses in GBM, we analyzed typical molecular mechanisms found in EMT. For example, transforming growth factor 1 (TGF-β1), a cytokine known to be a tumor suppression in early stage but an oncogenic factor in tumor growth in advances stages, can stimulate cancer cells to undergo EMT [17]. In this study, we investigated PIMT effects on EMT genes after TGF-β1 treatments. Despite the fact that U-87 MG cells are not epithelial cells, gene array analyses indicated that EMT genes were clearly regulated in glioma cells treated with TGF-β1. However, in U-87 MG cells, TGF-β1 did not affect PIMT gene and protein expression under conditions that regulated EMT markers. Importantly, PIMT silencing by siRNA inhibited in vitro migration of U-87 MG cells induced by TGF-β1. In contrast, overexpressed wild-type PIMT and TGF-β1 have additive effects on in vitro migration in U-87 MG cells. When PIMT was knocked down by siRNA, this blocked Slug induction by TGF-β1 while Snail stimulation by TGF-β1 was enhanced. As expected, overexpression of wild-type PIMT produced the opposite effects on Slug and Snail expression dependent on TGF-β1. On the other hand, N-cadherin and Fibronectin gene and protein levels were induced by TGF-β1, but PIMT overexpression in the presence of TGF-β1 affected only the Fibronectin protein level. Together, these results suggest that PIMT plays crucial functions in EMT responses dependent on TGF-β1 in glioma cells by regulating the expression of transcription factors such as Slug and Snail, which are critical players in EMT.

## 2. Results 

### 2.1. EMT Genes Are Regulated in U-87 MG Cells Treated with TGF-β1

**Figure 1:**
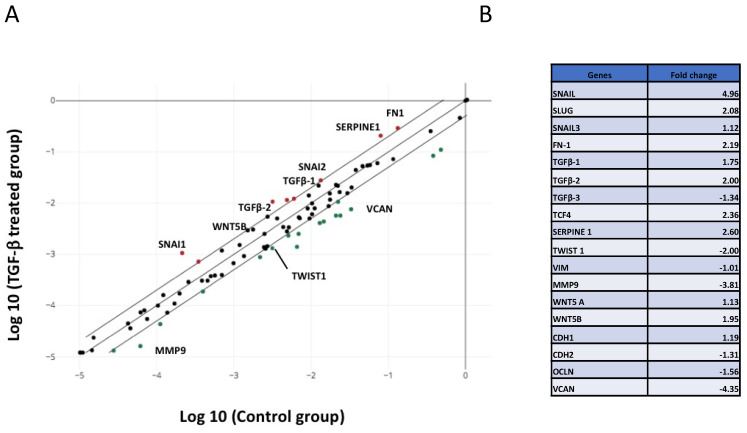
Gene array analysis demonstrated that EMT genes were regulated in U-87 MG cells treated with TGF-β1. Total RNA was isolated from U-87 MG cells treated, or not, with 10 ng/mL TGF-β1 for 24 h, then cDNA was synthesized. The Human EMT signaling pathways RT^2^ Profiler PCR Arrays served to identify EMT genes that were regulated following TGF-β1 treatment as described in the Materials and Methods section. (**A**) Graphic representation of the array showing gene expression levels. The values of upregulated genes are colored by red points, and those of downregulated genes are colored by green points. Data are delimited by external lines. Values higher than +2.00-fold and lower than −2.00-fold induction by TGF-β1 were considered significant. (**B**) Representative values of changes in EMT gene expression by TGF-β1 treatment in U-87 MG cells.

Since GBM are considered non-epithelial cells, the EMT process is called glial to mesenchymal transition (GMT) [18]. Here, we used U-87 MG cells, a common GBM model, and employed these cells with the Human EMT signaling pathways RT^2^ Profiler PCR Arrays to reveal which genes were regulated following TGF-β1 treatment. Total RNA was extracted from U-87 MG cells treated, or not, with 10 ng/mL TGF-β1 for 24 h, and cDNA was synthesized. The values of upregulated gene expression higher than +2.00-fold and those of downregulated gene expression lower than −2.00-fold by TGF-β1 were considered significant (Figure 1A). Among the examined EMT markers, the upregulated genes by TGF-β1were those for Snail, by 4.96-fold, Serpine1, by 2.60-fold, TCF4, by 2.36-fold, Fibronectin, by 2.19-fold, and Slug, by 2.08-fold (Figure 1A,B). The EMT markers that were down-regulated by TGF-β1 were genes for Versican, by 4.35-fold, MMP-9, by 3.81-fold, and Twist1, by 2.00-fold (Figure 1A,B). Together, these results showed that U-87 MG cells responded well to TGF-β1, and this could be an interesting glioma cell model to investigate PIMT functions in molecular mechanisms of TGF-β1 during EMT.

### 2.2. TGF-β1 Treatments Is Not Affecting PIMT Expression under Conditions That Induced EMT Markers in U-87 MG Cells 

**Figure 2:**
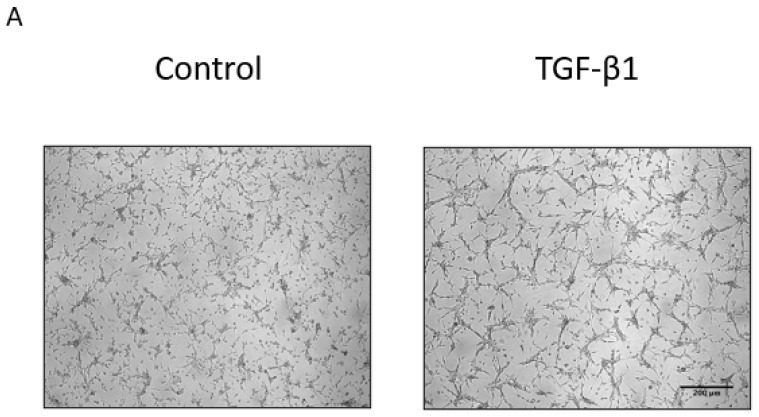

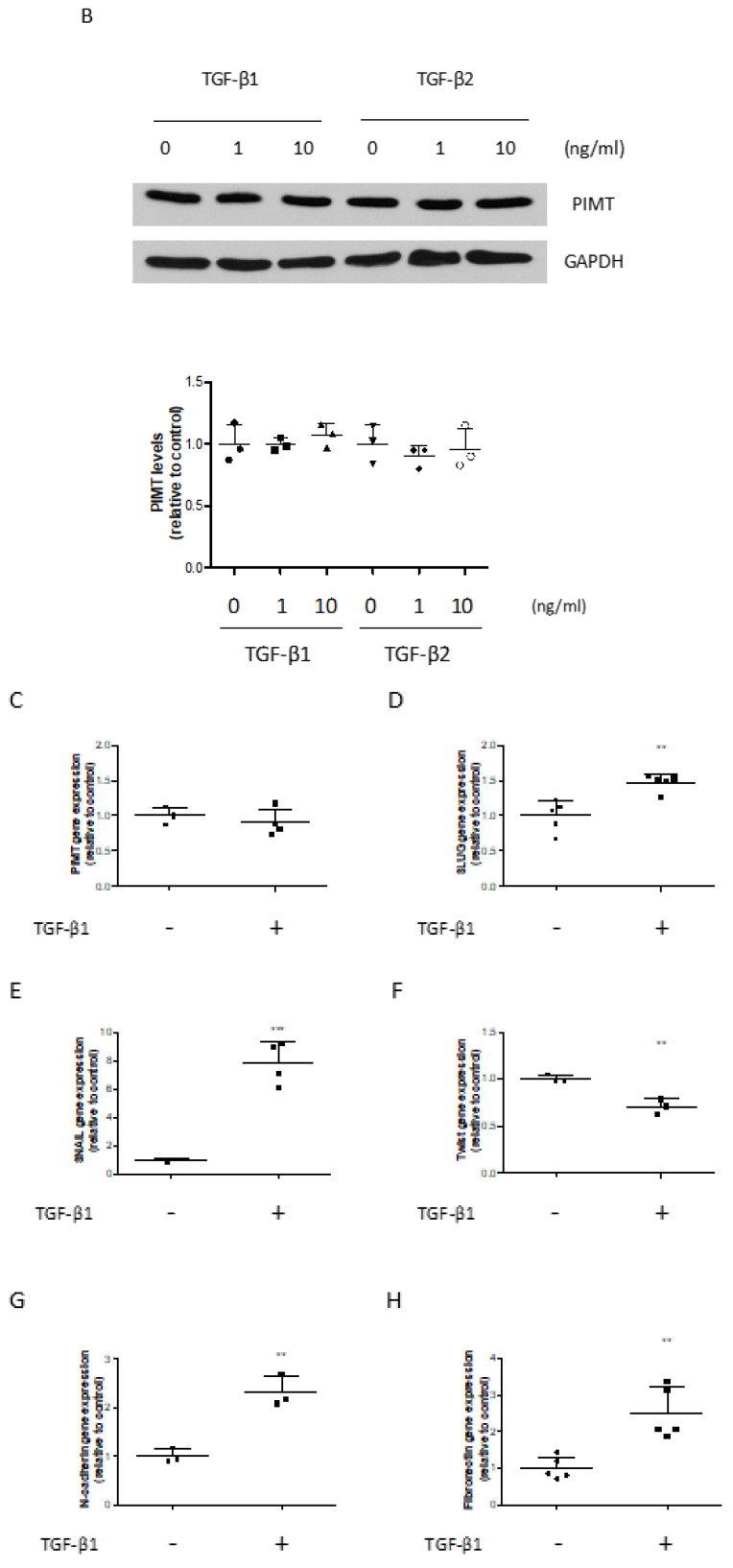

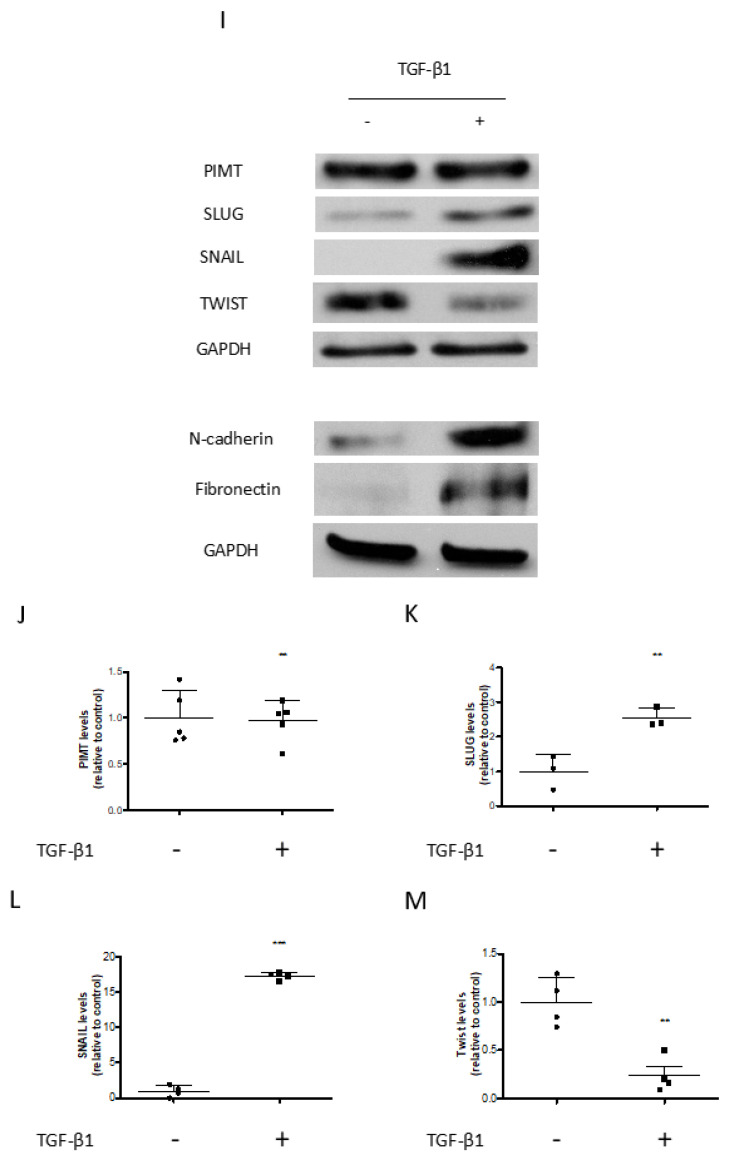

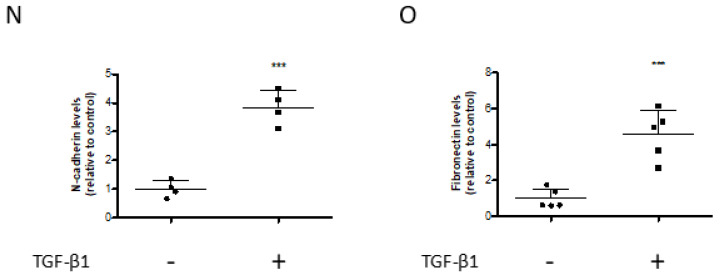
TGF-β1 did not influence PIMT expression under conditions that regulated EMT markers in U-87 MG cells. Cells were treated, or not, with 10 ng/mL TGF-β1 for 24 h. Representative effects of TGF-β1 on cell morphology were photographed in U-87 MG cells (**A**). The capacity of two TGF-β isoforms to modulate PIMT protein expression in U-87 MG cells was analyzed by Western blots using antibodies directed against PIMT and GAPDH as a loading control (**B**). Cells were treated with 0, 1 and 10 ng/mL of TGF-β1 and TGF-β2 for 24 h. Vertical scatter plots represent the ratio of immunodetected PIMT levels in treated cells to those in control cells calculated following densitometric analysis. Densitometric analysis of PIMT levels are representative of at least three independent experiments. Quantitative PCR analyses were performed to assess how 10 ng/mL TGF-β1 treatments for 24 h were regulating gene expression of EMT markers in U-87 MG cells (**C**–**H**). Furthermore, to evaluate the effects of TGF-β1 treatments on expression of EMT markers, proteins in cell lysates were separated by 12.5% polyacrylamide SDS-PAGE gels or in samples containing N-cadherin and Fibronectin by gradient of 4–20% polyacrylamide SDS-PAGE gels to improve protein separation. This was followed by Western blotting using antibodies directed against PIMT, EMT markers and GAPDH as a loading control (**I**). Densitometric quantifications were done (**J**–**O**). Again, vertical scatter plots represent the ratio of immunodetected protein levels in TGF-β1 treated cells to those in control cells calculated following densitometric analysis. Each column for quantitative PCR analyses and densitometric quantifications of Western blots is the mean ± SD from at least three independent experiments. Panels (**C**–**H**) and (**J**–**O**) are scatter plots analyzed with unpaired Student’s *t*-test indicating significantly different values, ** *p* < 0.01, and *** *p* < 0.001), from values between identified conditions. Scale bar = 200 μm.

Previously, we demonstrated that PIMT isoform 1 could regulate oncogenic features such as migration, invasion, and adhesion in U-87 MG glioma cells, and that this isoform was able to modulate cell morphology by remodelling F-actin and microtubule polymerization [10]. Other studies have reported that PIMT could regulate the expression of EMT markers in MDA-MB-231 breast cancer cells [7], in BUI-87 and SW780 bladder cancer cells [19], and in different lung adenocarcinoma cell lines [20]. Thus, these observations led us to investigate whether PIMT could be involved in EMT processes dependent on TGF-β1 when U-87 MG cells were treated with this cytokine. Interestingly, the shape of U-87 MG cells treated with 10 ng/mL TGF-β1 for 24 h converted these cells to star-shaped as compared to control cells (Figure 2A). Next, the effects of two TGF-β cytokines on PIMT expression in U-87 MG cells were analyzed by Western blots. PIMT protein levels when cells were treated with 0, 1 and 10 ng/mL of TGF-β1 and TGF-β2 for 24 h remained stable (Figure 2B). This lack of effect was also observed for longer treatments, up to 96 h, with both cytokines (data not shown). However, a treatment with 10 ng/mL TGF-β1 for 24 h modified U-87 MG cell morphology (Figure 2A). Thus, these conditions were kept in subsequent experiments to characterize expression of EMT markers. Initial experiments were performed using quantitative PCR analyses to assess gene expression of EMT markers in U-87 MG cells treated with TGF-β1. Similar to the PIMT protein, data showed that TGF-β1 did not affect PIMT isoform I gene expression (Figure 2C). As expected, TGF-β1 led to different regulation in the expression of EMT genes. Several gene levels were significantly upregulated, such as those for Slug by 1.5-fold (Figure 2D), Snail by 7.9-fold (Figure 2E), N-cadherin by 2.3-fold (Figure 2G), and Fibronectin by 2.5-fold (Figure 2H). However, the gene expression for Twist1 was significantly downregulated by 29% (Figure 2F). Then, Western Blots and densitometric evaluations were done to assess protein expression of these EMT markers when U-87 MG cells were treated with TGF-β1. Again, PIMT protein levels were unaffected by TGF-β1 (Figure 2I,J). However, the EMT proteins, as their genes, were regulated by TGF-β1 treatments (Figure 2I). The expression of Slug protein was significantly increased by 2.5-fold (Figure 2K), that of Snail protein by 17-fold (Figure 2L), that of N-cadherin protein by 3.9-fold (Figure 2N), and that of Fibronectin protein by 4.6-fold (Figure 2O). On the other hand, the protein level of Twist1 was significantly decreased by 76% upon TGF-β1 treatments (Figure 2M). Together, data obtained by Gene array analysis, quantitative PCR studies and Western blots demonstrated that TGF-β1 could induce EMT markers including Slug, Snail, N-cadherin and Fibronectin in U-87 MG cells, but that PIMT expression was unaffected at both mRNA and protein levels. 

### 2.3. PIMT Downregulation by siRNA Prevents In Vitro Migration of Human U-87 MG Cells Induced by TGF-β1

**Figure 3:**
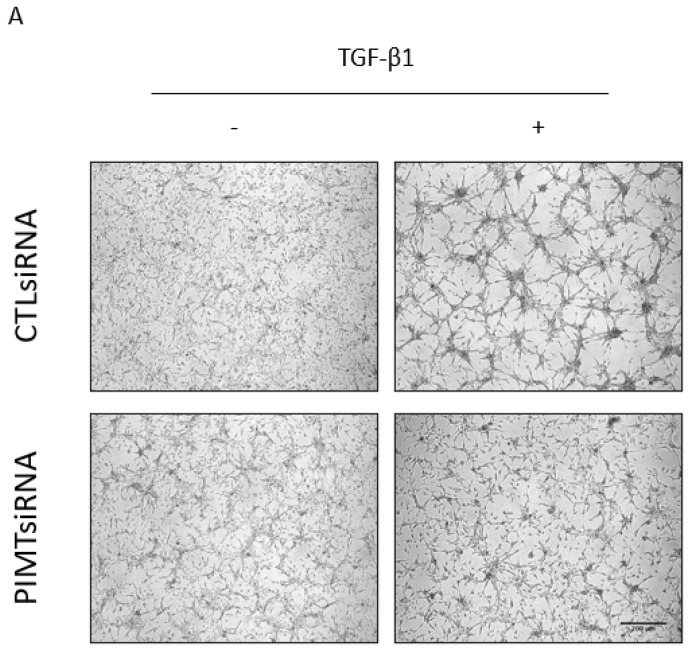

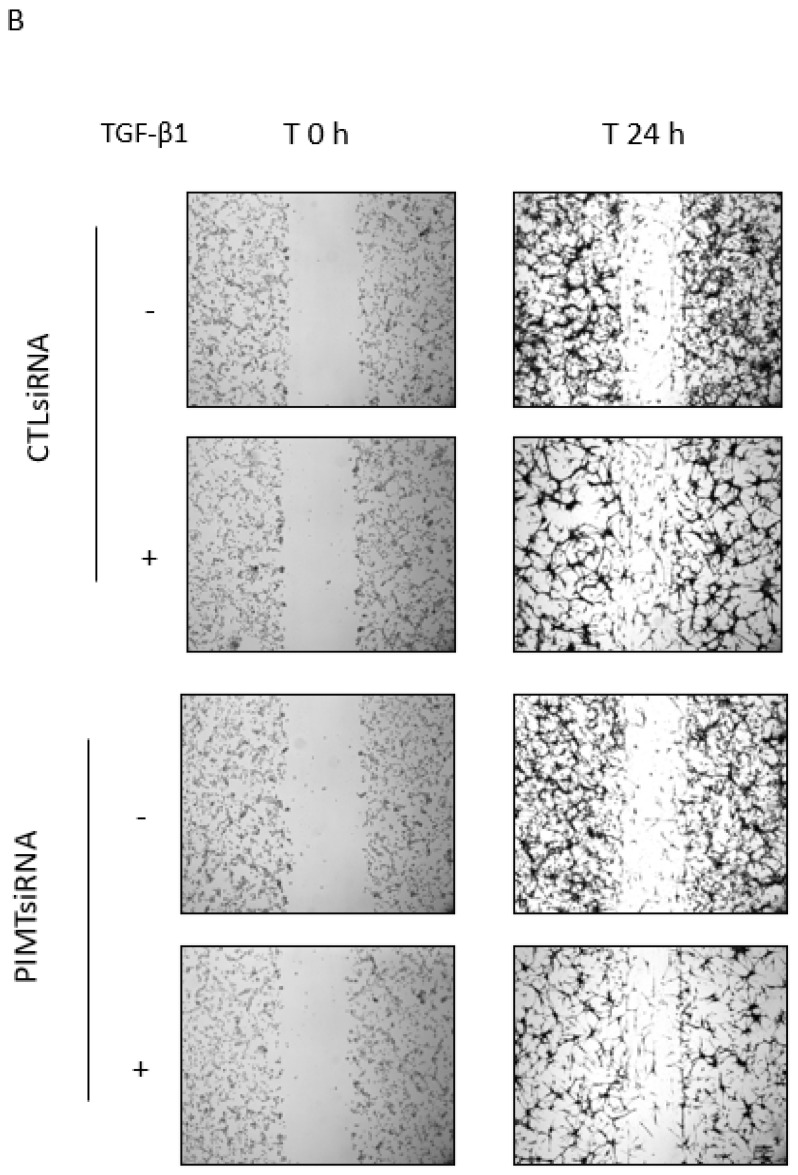

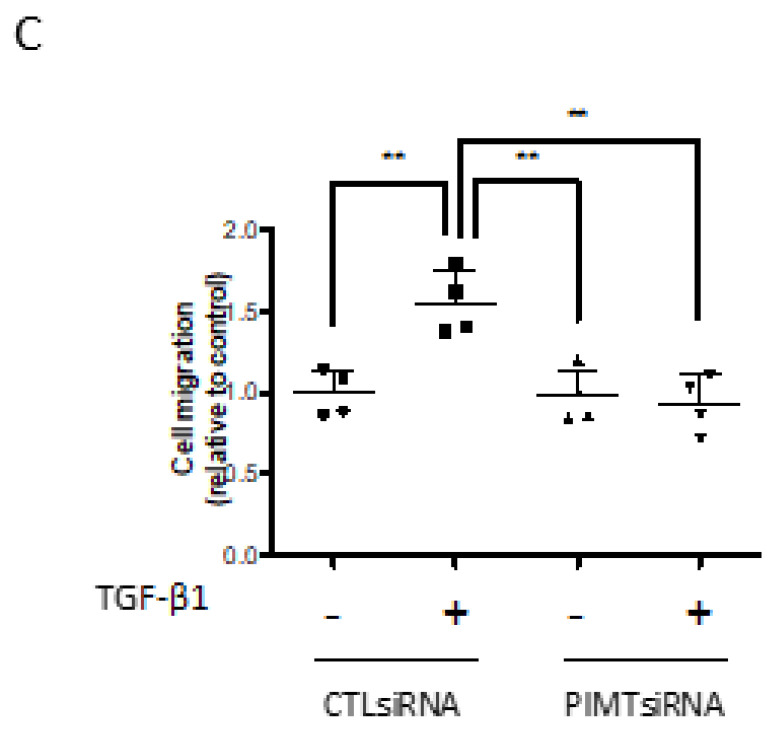
PIMT inhibition by siRNA prevented in vitro migration of human U-87 MG cells dependent on TGF-β1. Cells were transiently transfected with a PIMT siRNA or with AllStars negative control siRNA (Ctl siRNA) as described in the Materials and Methods section. Then, cells transfected with PIMT siRNA and control siRNA were treated, or not, with 10 ng/mL TGF-β1 for 24 h. Representative effects of TGF-β1 on morphology of cells transfected with PIMT siRNA and control siRNA were photographed (**A**). To study the effect of PIMT inhibition on cell migration, U-87 MG cells were transiently transfected with either PIMT siRNA or control siRNA, then wound-healing assays were performed. Cell migration was initiated by adding 10% FBS as a chemoattractant. Representative migration results for wound-healing assays at t = 0 and t = 24 h were photographed (**B**). However, at t = 0, cells were unstained while at t = 24 h, they were stained with 0.5% crystal violet to improve their visualization after migration. Wound closures were quantified, and panel (**C**) are vertical scatter plots analyzed with one-way ANOVA followed by the Bonferroni test indicating significantly different values, (** *p* < 0.01), from values between identified conditions. Each column is the mean ± SD from at least three independent experiments. Scale bar = 200 μm.

We reported that alterations in PIMT amounts acted on cytoskeleton organization, F-actin and microtubules, which were crucial components for regulating morphology and migration in U-87 MG cells [10]. In addition, we had already demonstrated the efficiency of PIMT siRNA treatments in U-87 MG cells to strongly inhibit PIMT protein expression [10]. Here, we tested whether PIMT silencing by siRNA could affect U-87 MG cell morphology and migration induced by TGF-β1. Interestingly, cells transfected with PIMT siRNA then treated with TGF-β1 underwent morphological changes that were less marked than those seen in cells transfected with control siRNA and treated with TGF-β1 (Figure 3A). Thus, these results suggested that PIMT was required to allow TGF-β1 to induce EMT responses in U-87 MG cells.

Migration of cancer cells is a crucial feature of glioma tumors. In connection with this, previously, we demonstrated that PIMT promoted migration and invasion in both U-87 MG and U-251 MG glioma cells [10]. Thus, we assessed whether PIMT was also involved in migration of U-87 MG cells treated with of TGF-β1. In in vitro wound-healing assays, the addition of 10 ng/mL TGF-β1 for 24 h significantly induced cell migration by 1.55-fold compared to cells untreated with TGF-β1 (Figure 3B,C). Importantly, PIMT downregulation by siRNA significantly blocked, by 40%, the migration of U-87 MG cells treated with TGF-β1 compared to cells transfected with control siRNA then treated with TGF-β1 (Figure 3B,C). In fact, when PIMT was inhibited by siRNA, migration of U-87 MG cells in the presence of TGF-β1 was similar to both to that in cells that were transfected with control siRNA and were untreated with TGF-β1 and to cells that were transfected with PIMT siRNA but were untreated with TGF-β1 (Figure 3B,C). These results from in vitro wound healing assays with PIMT depletion by siRNA when U-87 MG cells were treated by TGF-β1 underline that PIMT has an important role in TGF-β1 pathways that induced cell migration.

### 2.4. PIMT Knockdown by siRNA Blocks Slug Induction by TGF-β1 while Snail Stimulation by TGF-β1 Is Higher in Human U-87 MG Cells

**Figure 4:**
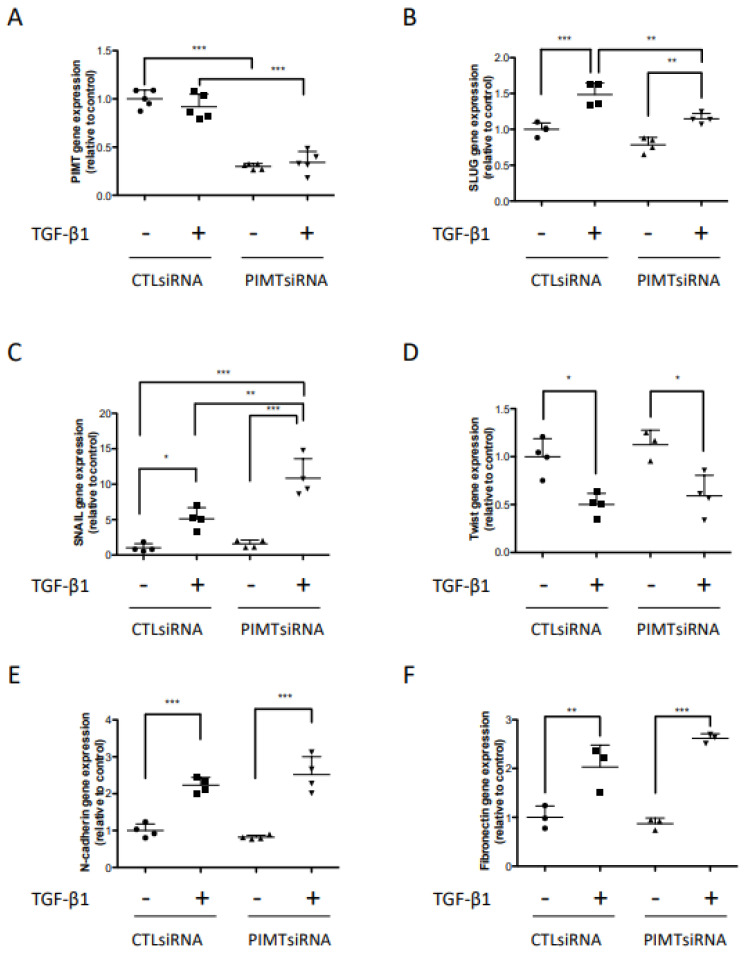

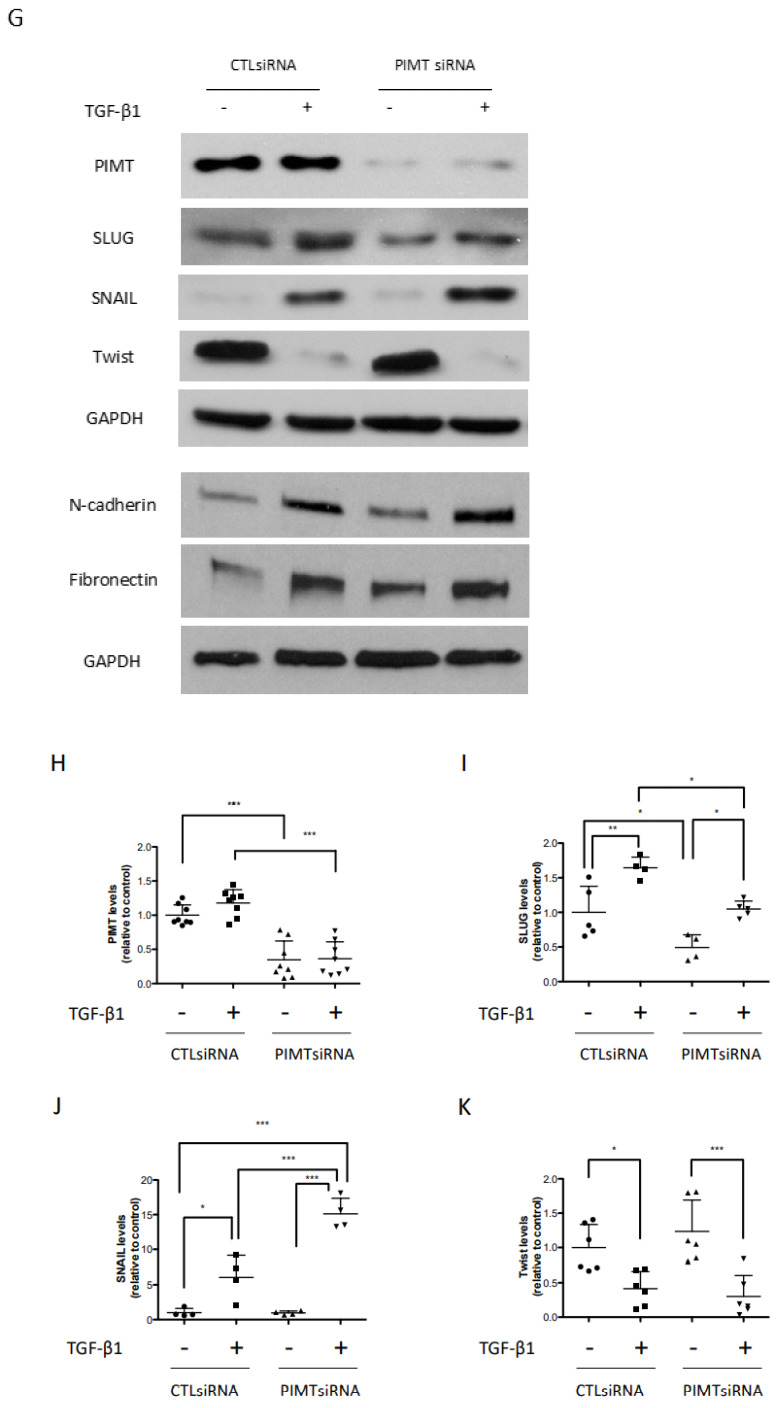

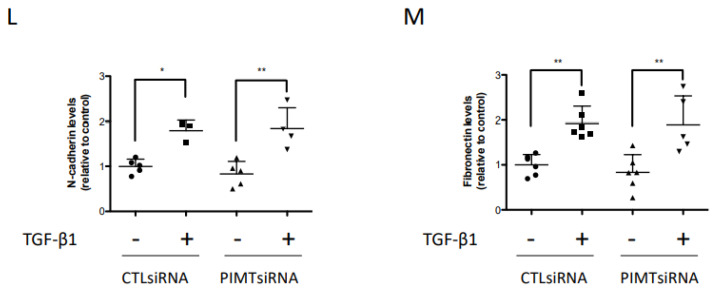
PIMT downregulation by siRNA blocked Slug induction by TGF-β1 but increased Snail stimulation by TGF-β1 in human U-87 MG cells. Cells were transiently transfected with a PIMT siRNA or with AllStars negative control siRNA (Ctl siRNA). Then, cells transfected with both siRNA were treated, or not, with 10 ng/mL TGF-β1 for 24 h. Quantitative PCR analyses were performed to investigate the impact of PIMT downregulation by siRNA on gene expression of PIMT and EMT markers following treatments of U-87 MG cells with TGF-β1 (**A**–**F**). Furthermore, to assess the efficiency of PIMT inhibition by siRNA, proteins in cell lysates were separated by 12.5% polyacrylamide SDS-PAGE gels or by gradient of 4–20% polyacrylamide SDS-PAGE gels to detect N-cadherin, and Fibronectin. This was followed by Western blotting using antibodies directed against PIMT, EMT markers and GAPDH as a loading control. Western blots showed protein levels at 72 h after cell transfection with control siRNA and PIMT siRNA (**G**). Densitometric quantifications (**H**–**M**) of protein levels were conducted to evaluate the effects of PIMT silencing by siRNA upon TGF-β1 treatments on protein expression of PIMT and EMT markers in U-87 MG cells. Vertical scatter plots represent the ratio of immunodetected protein levels in treated cells to those in their respective control cells calculated following densitometric analysis. Each column for quantitative PCR analyses (**A**–**F**) and densitometric quantifications of Western blots (**H**–**M**) is the mean ± SD from at least three independent experiments. Data in vertical scatter plots were analyzed with one-way ANOVA followed by the Bonferroni test indicating significantly different values, (* *p* < 0.05, ** *p* < 0.01, and *** *p* < 0.001), from values between identified conditions.

EMT is a critical step in regulating glioma cell migration and invasion [21]. The mRNA level and protein expression of EMT markers were examined by Real-Time PCR assay and Western blot analysis. As expected, PIMT mRNA levels in U-87 MG cells transfected by PIMT siRNA significantly decreased, by about 70%, in both the presence and the absence of TGF-β1 compared to their respective control cells (Figure 4A). Similar significant data were obtained for the inhibitory effect of PIMT siRNA on PIMT protein expression (Figure 4G,H). Interestingly, EMT markers were differently regulated by PIMT silencing. In the case of the transcription factor Slug, TGF-β1 treatments in cells transfected with control siRNA, significantly increased its gene expression by 49% (Figure 4B) while that of Slug protein was significantly higher by 65% (Figure 4G,I). Furthermore, addition of TGF-β1 to cells that were transfected with PIMT siRNA had a significant increase in Slug gene expression but this was less high, by 23% (Figure 4B), while the Slug protein level was significant higher, but only by 36% (Figure 4G,I). Importantly, PIMT inhibition by siRNA significantly blocked the TGF-β1 induction of Slug at both gene and protein levels compared to cells transfected with control siRNA and treated with TGF-β1 (Figure 4B,G,I). These levels remained similar to values observed in cells transfected with control siRNA and untreated with TGF-β1 (Figure 4B,G,I). In cells transfected with PIMT siRNA but without TGF-β1 treatment, Slug mRNA level was unaffected, but the protein level dropped significantly by 51% (Figure 4B,G,I) suggesting that PIMT could regulate basal level of Slug protein. 

The regulation of the transcription factor Snail was strong, since its gene expression was significantly stimulated by five-fold and that of its protein by six-fold in cells transfected with control RNA and treated with TGF-β1 as compared to control cells (Figure 4C,G,J). However, in contrast to Slug, Snail expression at both mRNA and protein levels were significantly enhanced by simultaneous PIMT inhibition by siRNA and TGF-β1 treatment compared to cells transfected with PIMT siRNA in the absence of TGF-β1 (Figure 4C,G,J). Consequently, Snail expression at both mRNA and protein levels were also significantly higher than those measured in cells transfected with control siRNA and treated with TGF-β1 (Figure 4C,G,J). The higher Snail expression when PIMT was silencing suggests that PIMT could interfere with the induction by TGF-β1. The final results were that in cells transfected with PIMT siRNA and treated with TGF-β1, Snail gene expression was significantly increased by 11-fold and that of its protein by 15-fold compared to U-87 MG cells transfected with control RNA and untreated with TGF-β1 (Figure 4C,G,J).

Unexpectedly, TGF-β1 significantly reduced mRNA amounts of the transcription factor Twist1 in both U-87 MG cells transfected with control siRNA, by 50%, and with PIMT siRNA, by 41%, as compared to their respective control cells (Figure 4D). Furthermore, the Twist1 protein levels were also significantly lower in cells transfected with control siRNA, by 59%, and with PIMT siRNA, by 70%, compared to their respective control cells (Figure 4G,K). On the other hand, PIMT silencing by siRNA did not affect Twist1 mRNA and protein levels in untreated and TGF-β1 treated cells compared to their respective control cells transfected with control siRNA (Figure 4D,G,K). N-cadherin is a transmembrane adhesion protein that plays a role in brain tumor invasion [22]. In cells transfected with the control siRNA, the addition of TGF-β1 significantly stimulated N-cadherin gene expression by 2.2-fold (Figure 4E). Under these conditions, the amount of N-cadherin protein significantly increased by 1.8-fold (Figure 4G,L). Similar results on gene and protein levels of N-cadherin were observed when cells were transfected with PIMT siRNA and underwent TGF-β1 treatments compared to cells untreated with the cytokine (Figure 4E,G,L). Furthermore, TGF-β1 addition to cells transfected with control siRNA or PIMT siRNA significantly induced, by 2.0-fold and 2.6-fold, Fibronectin mRNA expression compared to their respective controls (Figure 4F). Moreover, Western blot analyses conducted under these different conditions showed that TGF-β1 significantly increased Fibronectin protein levels by 1.9-fold in cells transfected with control siRNA and also by 1.9-fold in cells transfected with PIMT siRNA (Figure 4G,M).

### 2.5. PIMT and TGF-β1 Induce In Vitro Migration in U-87 MG Glioma Cells

**Figure 5:**
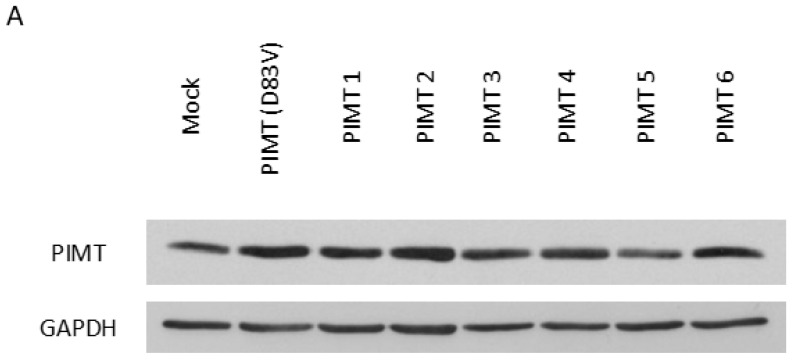

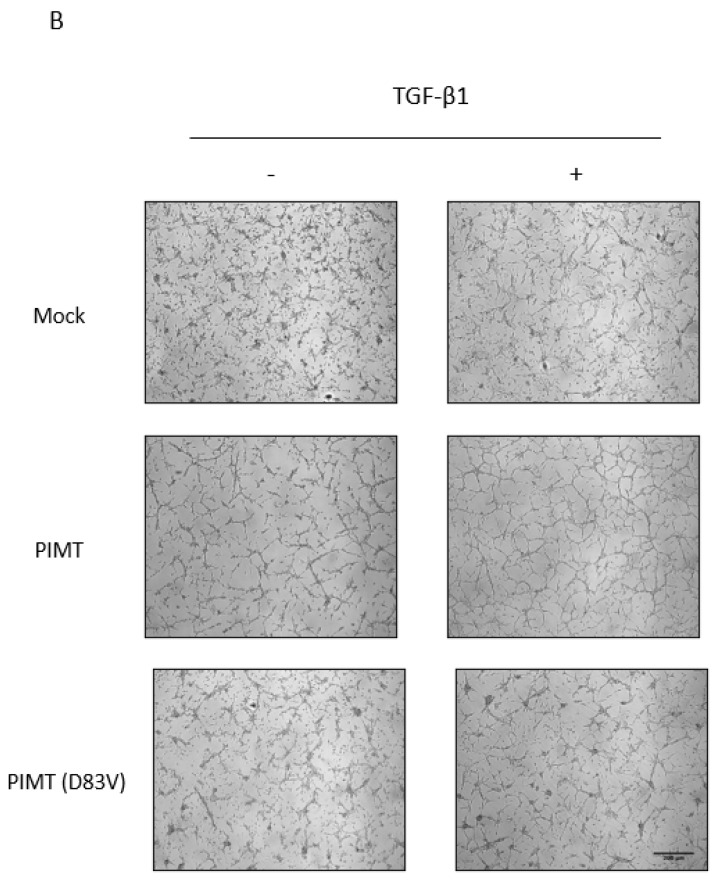

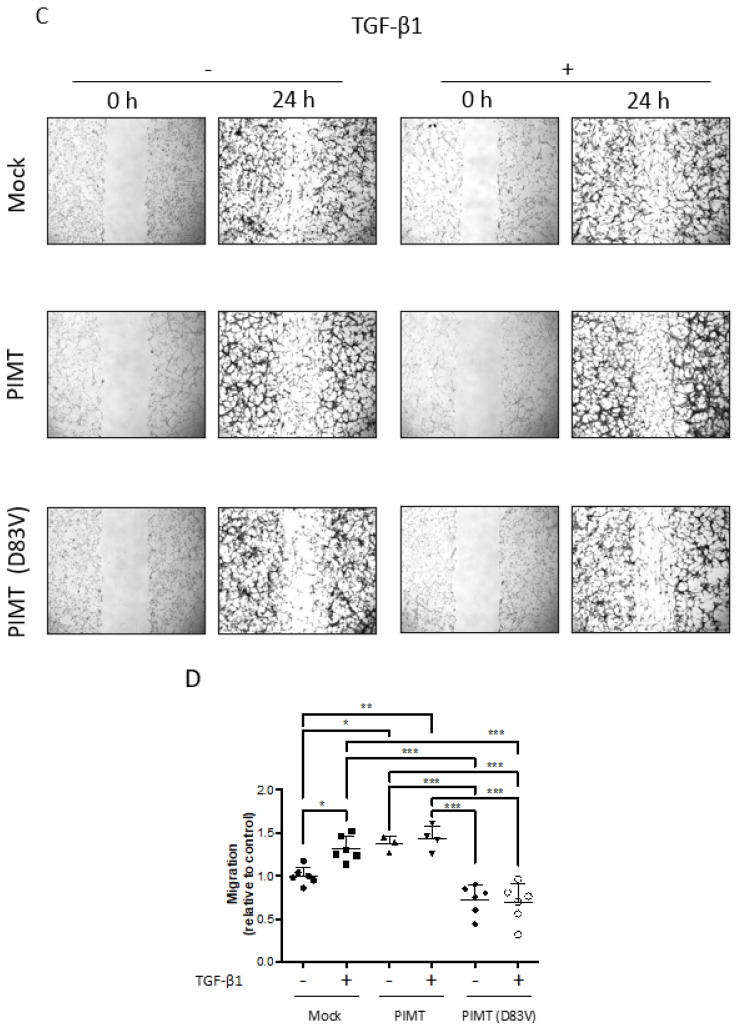
Both PIMT and TGF-β1 stimulated in vitro migration in U-87 MG glioma cells. Prior to investigating PIMT functions during cell migration induced by TGF-β1, we had to identify U-87 MG cell colonies overexpressing wild-type PIMT at the highest levels. Thus, several colonies of cells stably transfected with a *Pcmt1* gene-containing plasmid (pCMV6-PIMT) resulting in the overexpression of a wild-type PIMT were analyzed by Western blots. Comparative analysis with antibodies directed against PIMT and GAPDH in mock cells and in cells overexpressing PIMT identified the colony called PIMT2 as the one with the highest level of wild-type PIMT (**A**). Then, U-87 MG cells were stably transfected with the empty pCMV6 plasmid (Mock), the wild-type pCMV6-PIMT plasmid (PIMT), or the mutant pCMV6-PIMT(D83V) plasmid (PIMT D83V). These stable cell lines were treated, or not, with 10 ng/mL TGF-β1 for 24 h. Representative effects of TGF-β1 on morphology of cells overexpressing wild-type PIMT, mutant PIMT(D83V) and in mock cells were photographed (**B**). To study the effects of overexpression of wild-type PIMT and mutant PIMT(D83V) on cell migration, wound-healing assays were also performed. Representative migration patterns for U-87 MG cells in wound-healing assays at t = 0 and t = 24 h were photographed (**C**). The efficiency of migration invasion at t = 24 h were quantified (**D**). Data in vertical scatter plots were analyzed with one-way ANOVA followed by the Bonferroni test indicating significantly different values, (* *p* < 0.05, ** *p* < 0.01, and *** *p* < 0.001), from values between identified conditions. Each column is the mean ± SD from at least three independent experiments. Scale bar = 200 μm.

Results in Figure 3B,C showed that PIMT silencing by siRNA significantly blocked U-87 MG cell migration dependent on TGF-β1. Thus, we decided to further investigate the contribution of PIMT in U-87 MG cell migratory capacity stimulated by TGF-β1. In initial experiments, we selected U-87 MG cell colonies overexpressing wild-type PIMT at the highest levels. Western blot analysis allowed identification of the colony called PIMT2 as that with the highest level of wild-type PIMT (Figure 5A). Then, in subsequent experiments, we used the *Pcmt1* gene-containing plasmid (pCMV6-PIMT) isolated from this colony to establish stably transfected U-87 MG cells, which resulted in the overexpression of a wild-type isoform 1 of PIMT. Alternatively, cells were transfected with the empty plasmid pCMV6 or with a mutated *Pcmt1* gene-containing plasmid, the pCMV6-PIMT(D83V) plasmid expressing a mutant PIMT(D83V) isoform 1 of PIMT. This site of mutagenesis (D83) is a conserved residue involved in S-Adenosyl-L-methionine binding, the donor of methyl moiety [23]. Thus, a mutation in this sequence motif plays a disruptive role in methylation reactions dependent on PIMT. In contrast to the overexpression of wild-type PIMT isoform I, we previously showed that overexpression of mutated PIMT(D83V) did not enhance the migration and invasion of U-87 MG cells and U-251 MG glioma cells [10].

The effects of overexpressing wild-type PIMT and mutated PIMT(D83V) on U-87 MG cells morphology when untreated or treated with TGF-β1 were analyzed. The main effect on U-87 MG cell morphology was due to the overexpression of wild-type PIMT rather than the addition of TGF-β1 (Figure 5B). In fact, cells overexpressing wild-type PIMT were more elongated in the presence or the absence of TGF-β1 compared to mock cells (Figure 5B). However, when mutated PIMT(D83V) was overexpressed, U-87 MG cells kept an appearance that was similar to that seen in mock cells with a star-shaped morphology (Figure 5B). Next, the contribution of overexpressing wild-type PIMT on U-87 MG cell migration upon TGF-β1 treatment was examined. During in vitro wound healing assays in mock cells, addition of TGF-β1 significantly increased cell migration by about 32% (Figure 5C,D). As expected, overexpressing wild-type PIMT significantly enhanced U-87 MG cell migration, by 37%, compared to mock cells (Figure 5C,D). Moreover, a TGF-β1 treatment in cells that were overexpressing wild-type PIMT significantly stimulated the migration, by 43%, compared to cells transfected with the empty plasmid in the absence of TGF-β1 (Figure 5C,D). However, there was no marked difference between cells overexpressing wild-type PIMT and incubated, or not, with of TGF-β1 (Figure 5C,D). When cells were overexpressing mutant PIMT(D83V), the migration during wound healing assays in the absence of TGF-β1 was similar to that seen in mock cells and was significantly different from that measured in cells that were overexpressing wild-type PIMT (Figure 5C,D). Importantly, TGF-β1 was unable to induce migration when cells were overexpressing the mutant PIMT(D83V) compared to mock cells treated with TGF-β1 and mainly as compared to cells that overexpressed wild-type PIMT in the presence of TGF-β1 since in this case a significant inhibition of 52% was observed (Figure 5C,D). These results agreed with those where PIMT was silenced by siRNA, which prevented U-87 MG cell migration induced by TGF-β1 (Figure 3B,C). Together, these results demonstrate that PIMT expression and mainly its catalytic activity were crucial for U-87 MG migration since the overexpression of wild-type PIMT increased the migration while that of the mutated form of PIMT(D83V) blocked the TGF-β1 capacity to induce the migration.

### 2.6. Overexpression of Wild-Type PIMT Enhances Slug Expression but Lowers Snail Expression Dependent on TGF-β1 in Human U-87 MG Cells

**Figure 6:**
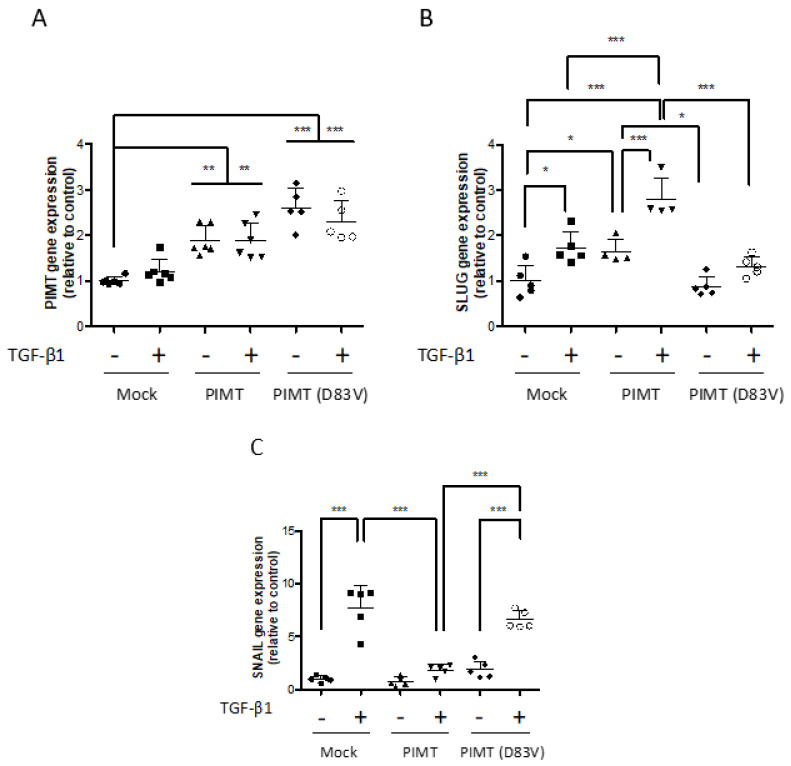

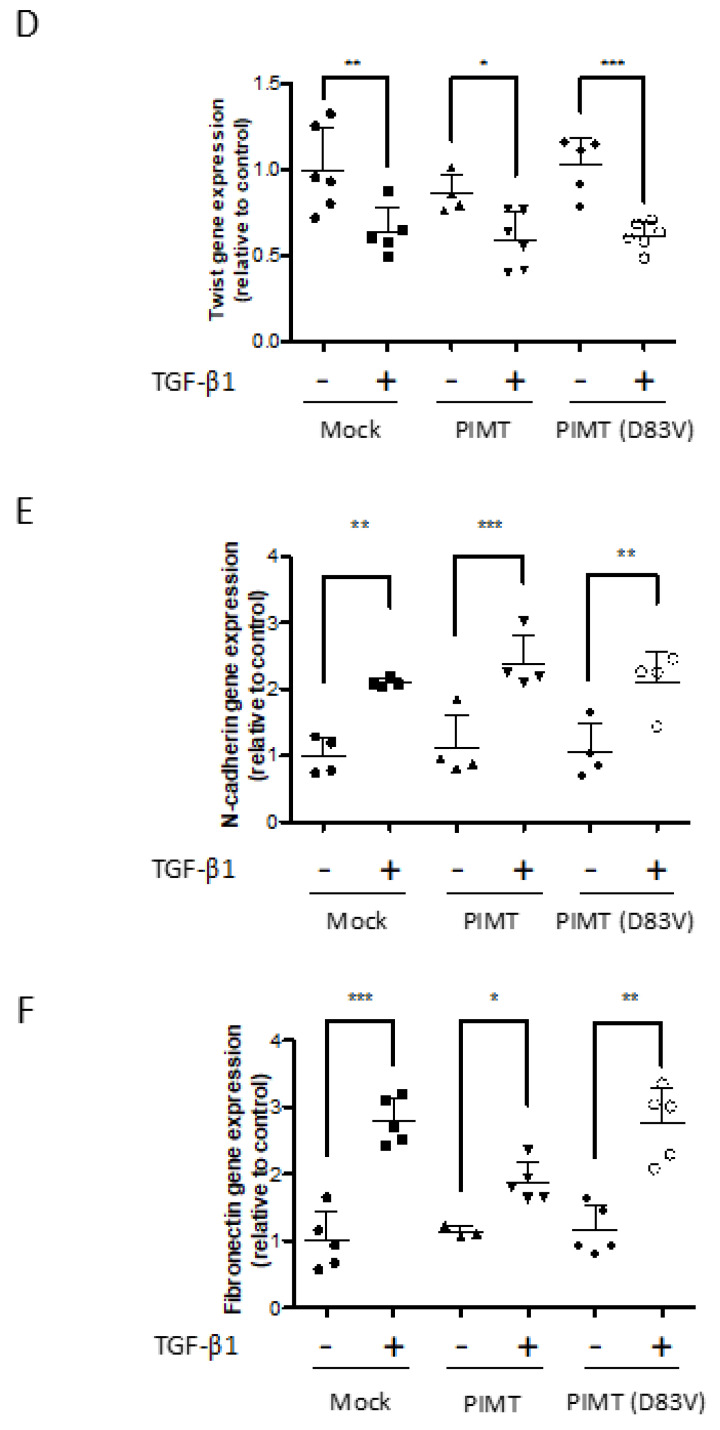

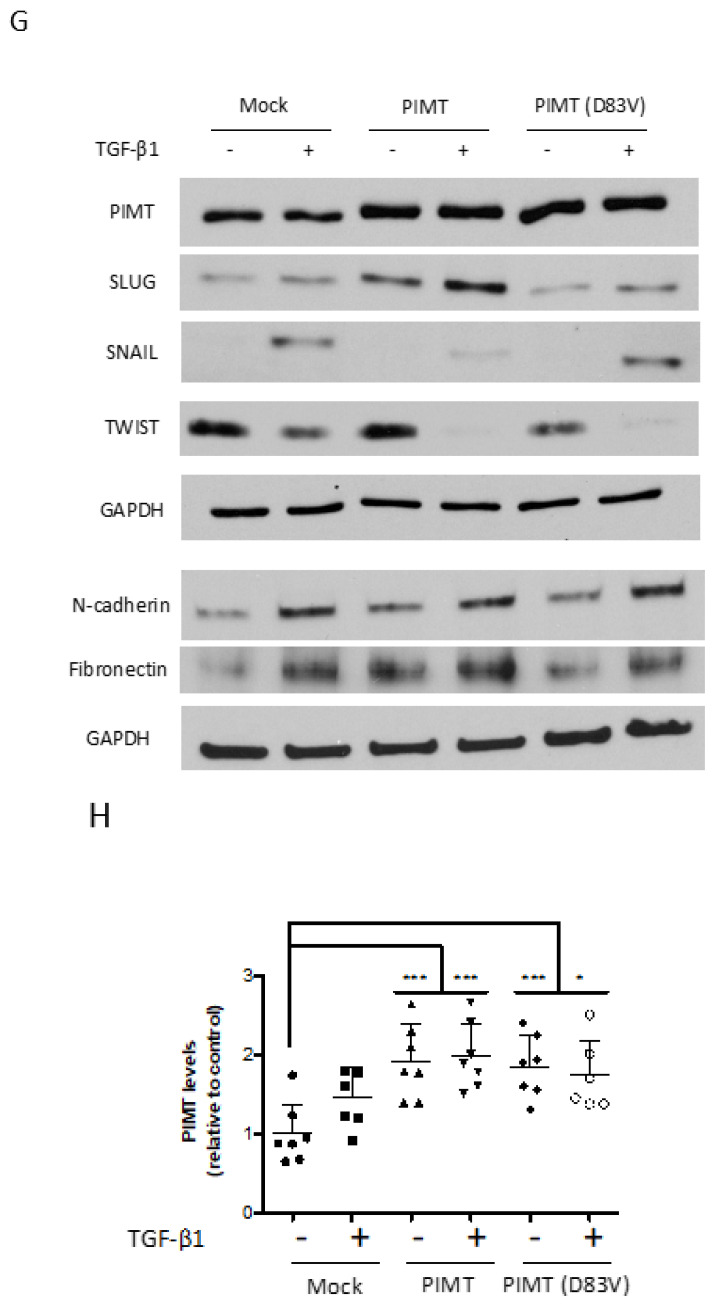

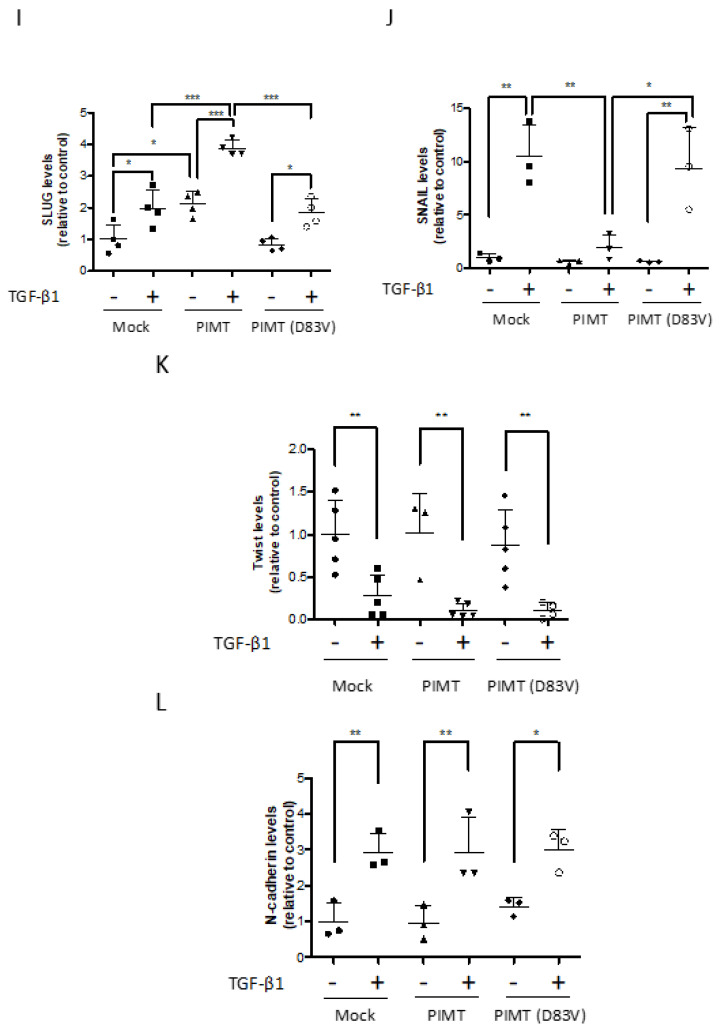

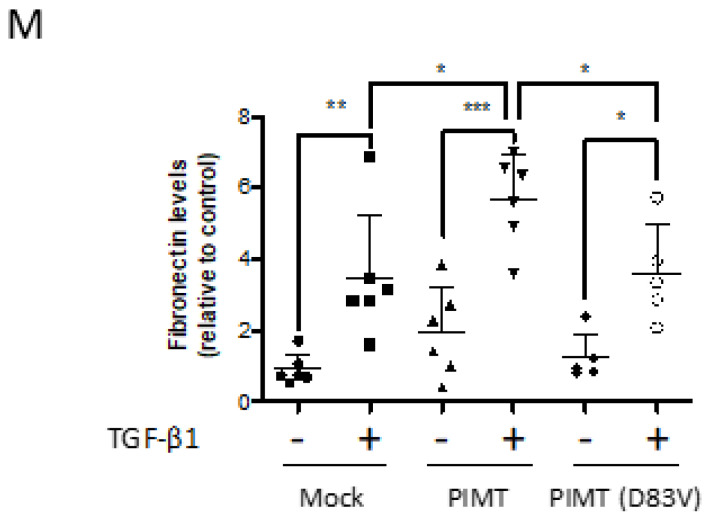
Analysis of the effects of overexpressing wild-type PIMT and mutant PIMT(D83V) on expression of EMT genes and EMT proteins upon TGF-β1 treatments in human U-87 MG cells. To analyze the contribution of PIMT on the expression of EMT markers upon TGF-β1 treatments, U-87 MG cells overexpressing both active and mutated PIMT were used. Thus, U-87 MG cells were stably transfected with the empty pCMV6 plasmid (Mock), the wild-type pCMV6-PIMT plasmid (PIMT), or the mutant pCMV6-PIMT(D83V) plasmid (PIMT D83V). Real-Time PCR assays were then performed to assess mRNA levels for PIMT and EMT markers when wild-type PIMT and mutant PIMT(D83V) were overexpressed and U-87 MG cells were treated, or not, with 10 ng/mL TGF-β1 for 24 h (**A**–**F**). Additionally, to evaluate how PIMT overexpression was acting on EMT marker proteins, cell lysates were separated by 12.5% polyacrylamide SDS-PAGE gels or by gradient of 4–20% polyacrylamide SDS-PAGE gels. This was followed by Western blotting using antibodies directed against PIMT, EMT markers and GAPDH as a loading control (**G**). Densitometric quantifications (**H**–**M**) of protein levels were also done to measure the effects of PIMT overexpression during TGF-β1 treatments on protein expression of PIMT and EMT markers in U-87 MG cells. Vertical scatter plots represent the ratio of immunodetected protein levels in TGF-β1 treated cells or overexpressing PIMT to those in their respective control cells calculated following densitometric analysis. Each column seen in scatter plots for quantitative PCR analyses (**A**–**F**) and densitometric quantifications of Western blots (**H**–**M)** the mean ± SD from at least three independent experiments. Data in scatter plots were analyzed with one-way ANOVA followed by the Bonferroni test indicating significantly different values, (* *p* < 0.05, ** *p* < 0.01, and *** *p* < 0.001), from values between identified conditions.

Since PIMT silencing differently affected the expression of EMT markers regulated by TGF-β1 added to U-87 MG cells (Figure 4), we decided to investigate, by Real-Time PCR assays and Western blot analyses, the consequences of PIMT overexpression on these EMT markers. In the absence of TGF-β1, stable cell lines that were transfected with the wild-type pCMV6-PCMT1 plasmid, the PIMT mRNA level was significantly increased by 1.9-fold, while in cells transfected with the inactive mutant pCMV6-PCMT1(D83V) plasmid, the PIMT mRNA amount was significantly higher, by 2.6-fold, compared to mock cells (Figure 6A). However, the addition of TGF-β1 did not increase PIMT mRNA levels in the three stables cell lines since they were similar to those measured in the absence of TGF-β1 (Figure 6A). Similar results were observed for the lack of effect of TGF-β1 on PIMT protein expression in cells that overexpressed wild-type PIMT and mutant PIMT(D83V) (Figure 6G,H). Although wild-type PIMT isoform I was overexpressed, which means that the enzyme was about twice more abundant in cells, PIMT mRNA and protein levels were unregulated by TGF-β1 in U-87 MG cells.

Indeed, TGF-β1 treatments significantly increased gene expression of the transcription factor Slug in mock cells (1.7-fold), in cells overexpressing wild-type PIMT (2.8-fold), but to a lesser degree in cells overexpressing mutant PIMT(D83V) (1.3-fold) compared to their respective controls (Figure 6B). In the absence of TGF-β1, overexpression of wild-type PIMT significantly stimulated Slug gene expression by 1.7-fold (Figure 6B). Thus, PIMT was able to upregulate the basal expression of Slug gene. This assumption was confirmed by data obtained from cells overexpressing mutant PIMT(D83V) in the absence of TGF-β1 because, in this case, Slug gene expression was similar to that in mock cells and mainly its value was significantly lower, by about 47%, relative to that seen in cells that were overexpressing wild-type PIMT (Figure 6B). Since TGF-β1 and wild-type PIMT were independently able to stimulate Slug gene expression, the result of adding TGF-β1 to cells overexpressing wild-type PIMT was to significantly enhance Slug gene expression by 2.8-fold compared to mock cells untreated with TGF-β1 (Figure 6B). The addition of TGF-β1 to cells overexpressing wild-type PIMT also led to a significant increase of Slug gene expression, by 1.6-fold, compared to mock cells treated with TGF-β1, respectively (Figure 6B). Similar significant stimulations were observed by Western blots and densitometric quantifications on the expression of Slug protein following TGF-β1 treatments of mock cells, cells overexpressing wild-type PIMT and again to a lower extent in cells overexpressing mutant PIMT(D83V) (Figure 6G,I). Together, these data suggested that higher Slug gene and protein levels in the presence of TGF-β1 in U-87 MG cells overexpressing wild-type PIMT isoform I resulted from the additive effects of two factors: first, a stimulation by wild-type PIMT on basal expression of Slug gene and protein, and second an induction by TGF-β1 treatments.

As expected, the Snail mRNA level was strongly and significantly upregulated, by 7.7-fold, in mock cells treated with TGF-β1 (Figure 6C). However, TGF-β1 treatment did not induce Snail gene expression significantly in cells overexpressing wild-type PIMT (Figure 6C). Conversely, in cells overexpressing mutant PIMT(D83V), the ability of TGF-β1 to induce Snail gene expression was significant elevated, by 6.6-fold (Figure 6C). Furthermore, adding TGF-β1 to cells overexpressing mutant PIMT(D83V) was even significantly better comparatively to cells overexpressing wild-type PIMT and treated with TGF-β1 since the induction was by 3.7-fold between these cells (Figure 6C). At protein levels, TGF-β1 treatment triggered similar responses in Snail expression, as seen before at mRNA levels, when experiments were performed in mock cells, in cells overexpressing wild-type PIMT and in cells overexpressing mutant PIMT(D83V) (Figure 6G,J). Consequently, Snail gene and protein levels in cells overexpressing wild-type PIMT in the presence of TGF-β1 were significantly lower than those measured in mock cells and in cells overexpressing mutant PIMT(D83V) (Figure 6C,G,J). The lower Snail gene and protein levels when wild-type PIMT was overexpressed suggest that this enzyme was interfering with Snail induction by TGF-β1. 

In the case the transcription factor Twist1, its gene expression in U-87 MG cells overexpressing wild-type PIMT and mutant PIMT(D83V) remained unaffected in the absence of TGF-β1 compared to mock cells (Figure 6D). Thus, PIMT did not appear to regulate the basal gene expression of Twist1. When TGF-β1 was added, significant changes were noted with about 40% inhibition in mock cells, in cells overexpressing PIMT and those overexpressing mutant PIMT(D83V) compared to their respective control cells untreated with TGF-β1 (Figure 6D). At proteins levels, similar results were observed with TGF-β1 treatments. In mocks cells, in cells overexpressing wild-type PIMT and mutated PIMT(D83V), the expression of Twist1 level was lower, between 72% and 89%, compared to control cells untreated with TGF-β1 (Figure 6G,K). 

In U-87 MG cells overexpressing wild-type PIMT and in those overexpressing mutant PIMT(D83V), the N-cadherin and Fibronectin gene expressions were similar to those observed in mock cells in the absence of TGF-β1 (Figure 6E,F). The addition of TGF-β1 significantly stimulated N-cadherin mRNA levels in mock cells by about 2.1-fold, and in cells that overexpressed wild-type PIMT by about 2.4-fold, while it was by about 2.1-fold in cells transfected with mutant PIMT(D83V) (Figure 6E). Thus, TGF-β1 in U-87 MG cells acted independently of PIMT amount or activity. Similarly, TGF-β1 significantly increasing Fibronectin gene expression by 2.8-fold in mock cells, by 1.9-fold in cells that overexpressed wild-type PIMT and by 2.8-fold in cells transfected with mutant PIMT(D83V) (Figure 6F). At protein levels, N-cadherin amounts upon treatments with TGF-β1 significantly increased by about 2.9-fold in mock cells, and by about 2.9-fold in cells that overexpressed wild-type PIMT, while it was increased by about 3-fold in cells transfected with mutant PIMT(D83V) (Figure 6G,L). However, TGF-β1 significantly induced Fibronectin protein levels by 3.5-fold in mock cells and by 3.6-fold in cells transfected with mutant PIMT(D83V) (Figure 6G,M). Interestingly, in cells that overexpressed wild-type PIMT in the presence of TGF-β1, the Fibronectin protein level was significantly induced compared to cells that overexpressed wild-type PIMT but untreated with TGF-β1 (Figure 6G,M) resulting in a pronounced 5.7-fold induction relatively to mock cells untreated with TGF-β1 (Figure 6G,M). Moreover, in cells overexpressing wild-type PIMT and treated with TGF-β1, the Fibronectin protein level was significantly higher compared to both those quantified in mock cells and in cells overexpressing mutated PIMT(D83V) that were treated with TGF-β1 (Figure 6G,M).

### 2.7. PIMT Silencing by siRNA of Overexpressed Wild-Type PIMT in U-87 MG Cells Blocks the Higher Slug Expression and Prevents the Lower Snail Expression Dependent on TGF-β1

**Figure 7:**
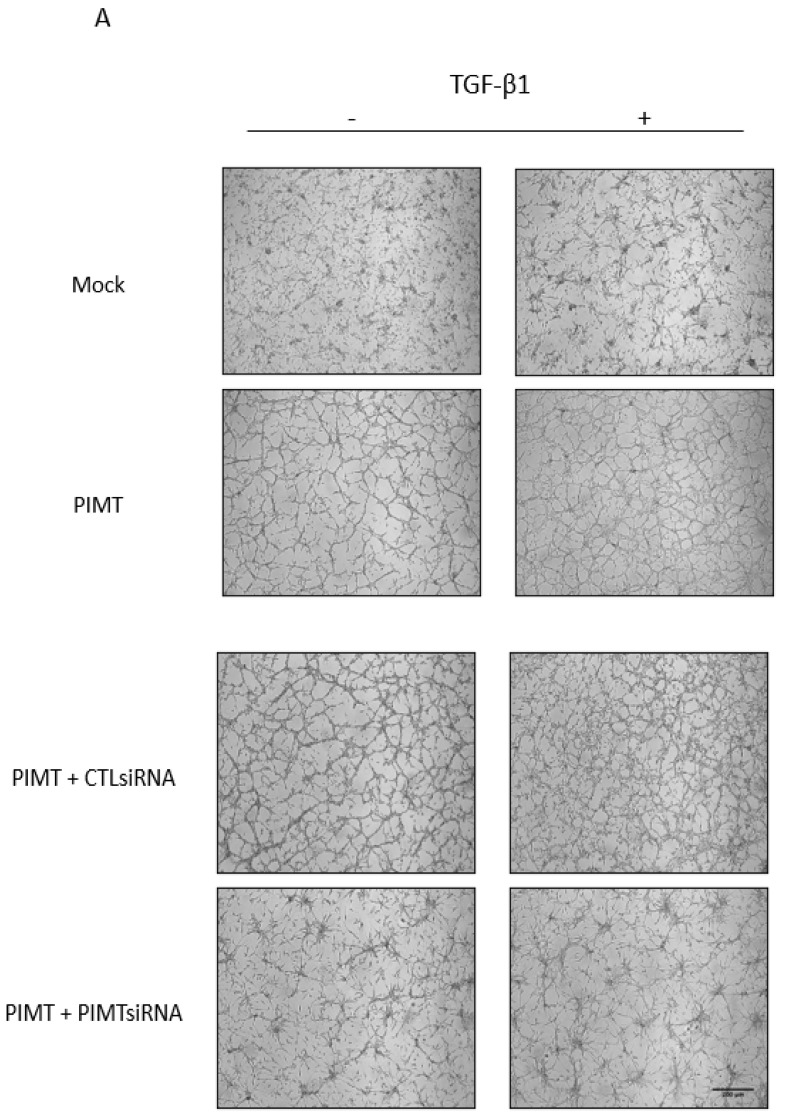

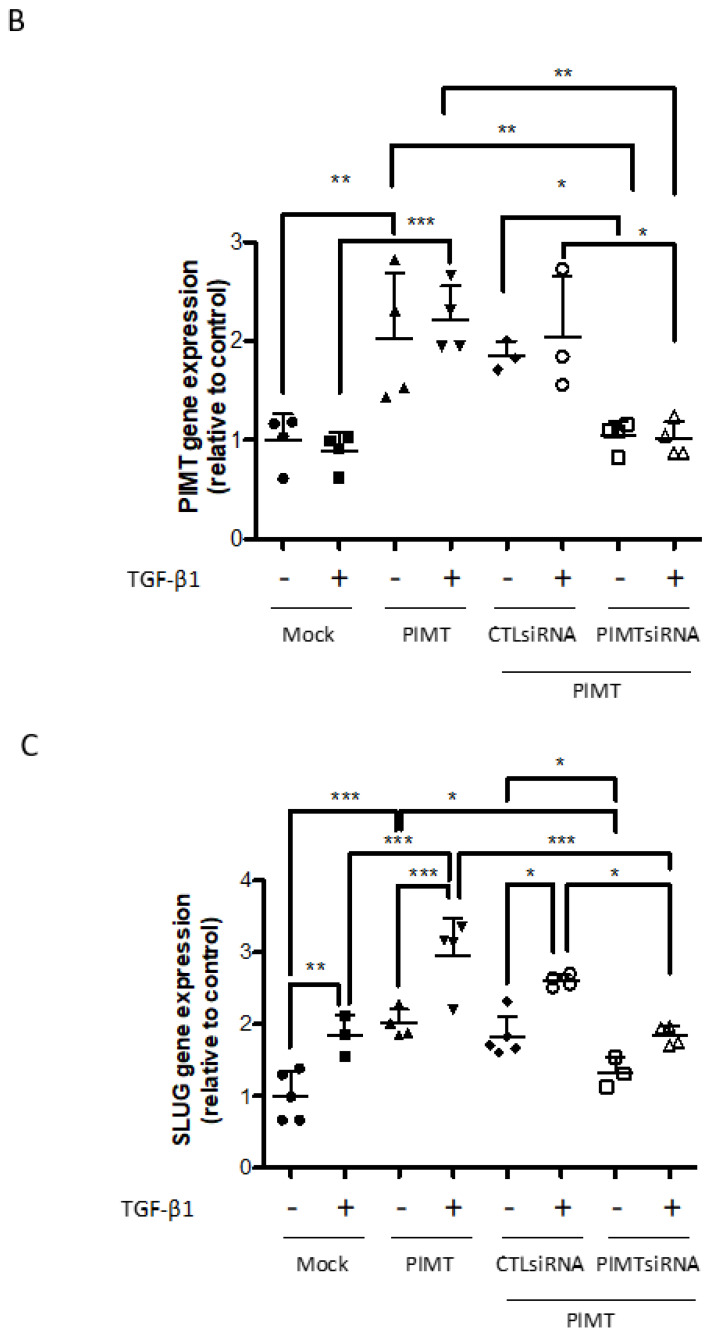

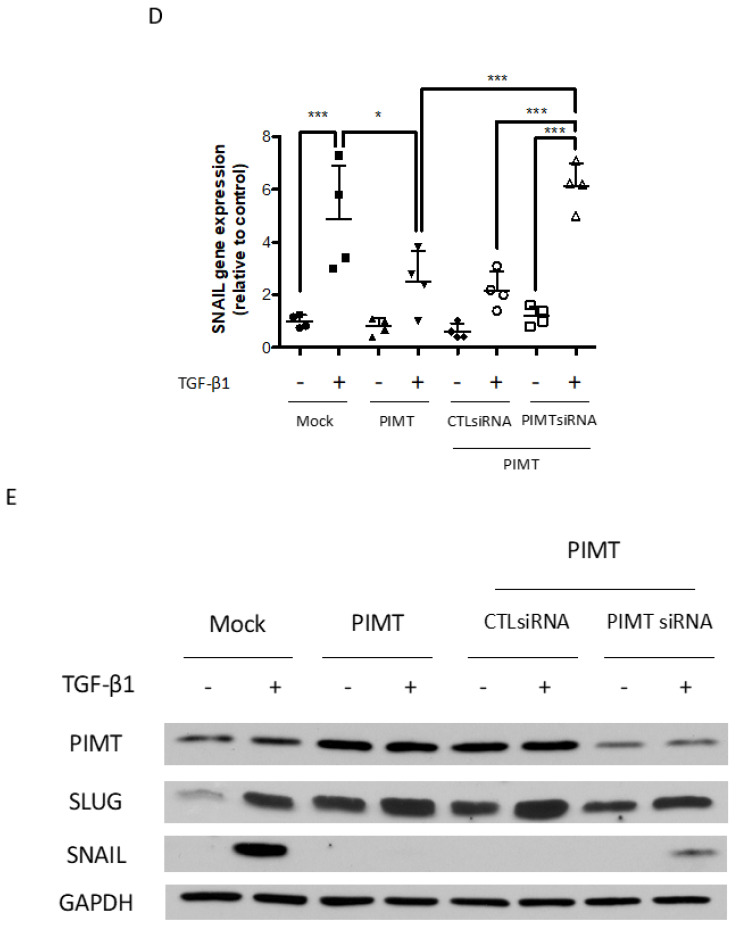

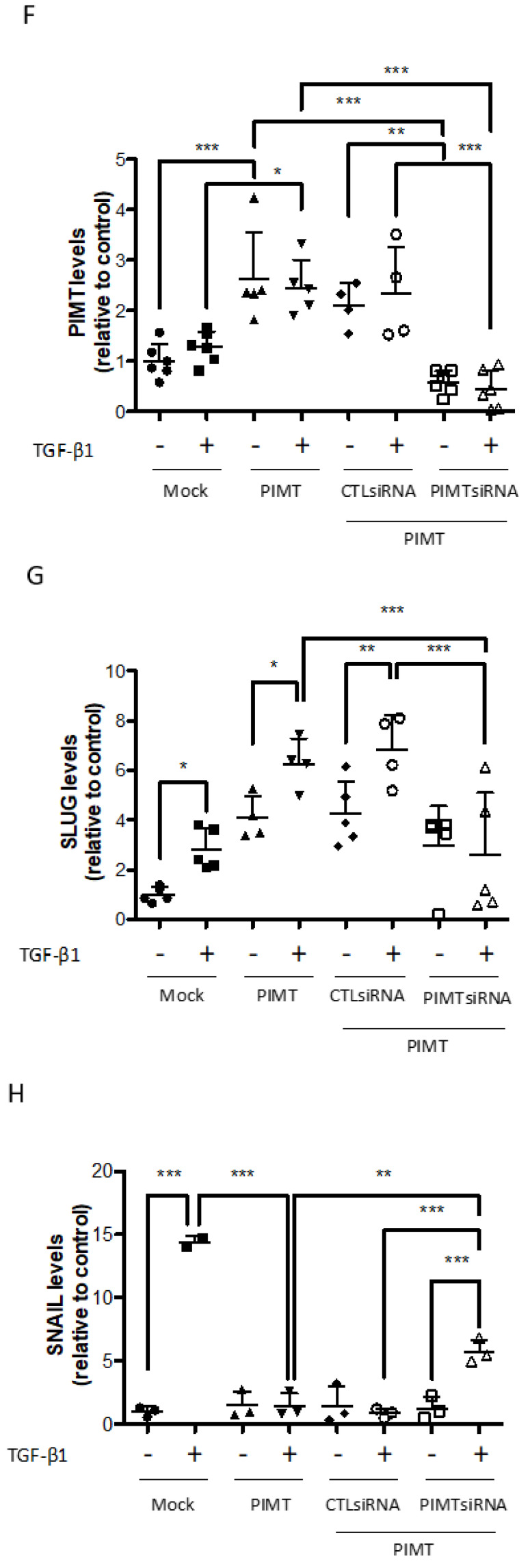
PIMT blockade by siRNA in U-87 MG cells overexpressing wild-type PIMT prevented both higher Slug gene and protein expression and lower Snail gene and protein expression regulated by TGF-β1. To support data that U-87 MG cells overexpressing mutant PIMT(D83V) did not manifest the same potential as cells overexpressing wild-type PIMT to regulate Slug and Snail gene expression dependent on TGF-β1, complementary experiments were conducted. Thus, U-87 MG cells were stably transfected with the empty pCMV6 plasmid (Mock) or the wild-type pCMV6-PIMT plasmid (PIMT). Subsequently, stable cell lines overexpressing wild-type PIMT were transiently transfected with a PIMT siRNA or with AllStars negative control siRNA (Ctl siRNA). Finally, mock cells, cells overexpressing wild-type PIMT, cells overexpressing wild-type PIMT and transfected with control siRNA, and cells overexpressing wild-type PIMT and transfected with PIMT siRNA were incubated, or not, with 10 ng/mL TGF-β1 for 24 h. Representative effects of TGF-β1 on morphology in mock cells and in cells overexpressing wild-type PIMT under different experimental conditions were photographed (**A**). Quantitative PCR analyses were performed to measure the impact of downregulating overexpressed wild-type PIMT by siRNA on gene expression of PIMT, Slug and Snail when U-87 MG cells were incubated, or not, with 10 ng/mL TGF-β1 for 24 h (**B**–**D**). Furthermore, these effects of PIMT inhibition by siRNA in cells overexpressing wild-type PIMT were also analyzed upon TGF-β1 treatment on expression of Slug and Snail proteins. Proteins were revealed by Western blot using antibodies directed against PIMT, Slug, Snail and GAPDH as used as a loading control (**E**). Densitometric quantifications of PIMT, Slug and Snail protein levels (**F**–**H**) were performed to evaluate the impact of PIMT inhibition by siRNA in U-87 MG cells overexpressing PIMT upon TGF-β1 treatments. Vertical scatter plots provide the ratio of immunodetected protein levels in TGF-β1 treated cells or overexpressing PIMT under various conditions to those in their respective control cells calculated following densitometric analysis. Each column seen in scatter plots for quantitative PCR analyses (**B**–**D**) and densitometric quantifications of Western blots (**F**–**H**) are the mean ± SD from at least three independent experiments. Data in scatter plots were analyzed with one-way ANOVA followed by the Bonferroni test indicating significantly different values, (* *p* < 0.05, ** *p* < 0.01, and *** *p* < 0.001), from values between identified conditions. Scale bar = 200 μm.

Results with overexpression of wild-type PIMT isoform I in U-87 MG glioma cells showed that the active enzyme was involved in pathways dependent on TGF-β1 to regulate both Slug and Snail gene and protein expressions (Figure 6). However, these effects on Slug and Snail gene and protein expressions were lower or absent when U-87 MG cells were overexpressing mutant PIMT(D83V) (Figure 6). To support these observations, complementary experiments were performed in U-87 MG cells overexpressing wild-type PIMT, but this time the high amount of active enzyme was partially silenced by using PIMT siRNA. In cells overexpressing wild-type PIMT then transfected with PIMT siRNA, their morphology was strongly modified compared to cells overexpressing wild-type PIMT and to cells overexpressing wild-type PIMT then transfected with control siRNA (Figure 7A). In fact, their morphology looked similar to that observed in mock cells (Figure 7A). Upon TGF-β1 treatments, the differences in cell morphologies were less marked but effects of PIMT silencing by siRNA in cells overexpressing wild-type PIMT compared to cells overexpressing wild-type PIMT and to cells overexpressing wild-type PIMT then transfected with control siRNA, were still obvious (Figure 7A).

Next, Real-Time PCR assays were done to determine the effects of inhibiting wild-type PIMT on PIMT, Slug and Snail gene expression. As expected, overexpression of wild-type PIMT significantly increased PIMT mRNA levels by about 2.0-fold in the absence of TGF-β1 and by 2.2-fold in the presence of TGF-β1 (Figure 7B). When stable cells overexpressing wild-type PIMT were transfected with PIMT siRNA, these higher mRNA levels were significantly reduced compared to those measured in cells overexpressing wild-type PIMT in the absence (by 49%) and the presence (by 54%) of TGF-β1 (Figure 7B), and they were almost similar to those seen in mock cells in the absence and the presence of TGF-β1 (Figure 7B). Indeed, PIMT mRNA levels were significantly lower in cells transfected with PIMT siRNA compared to those transfected with control siRNA (Figure 7B). In addition, similar results at PIMT proteins levels were observed (Figure 7E,F). For example, PIMT protein levels were significantly higher by 2.6 and 2.5-fold in cells that overexpressed wild-type PIMT and were treated, or not, with TGF-β1 compared to their respective mock cells (Figure 7E,F). However, when cells overexpressing wild-type PIMT were also transfected with PIMT siRNA, PIMT protein levels significantly decreased by 78% to 82% in TGF-β1 treated and untreated cells compared to cells overexpressing wild-type PIMT that were treated or not with TGF-β1 (Figure 7E,F). Thus, PIMT siRNA treatments were efficient in blocking PIMT overexpression and thus potentially mimicking responses previously found on Slug and Snail expression when U-87 MG cells were overexpressing mutant PIMT(D83V) in the presence of TGF-β1.

As previously noted in Figure 6B, overexpression of wild-type PIMT significantly stimulated Slug gene expression in the absence of TGF-β1 (Figure 7C). As expected, this Slug gene induction in the absence of TGF-β1 was significantly blocked when cells overexpressing wild-type PIMT were transfected with PIMT siRNA (Figure 7C). TGF-β1 addition to mock cells and to cells overexpressing wild-type PIMT induced significant increase in Slug gene expression, by about 1.8 and 3-fold, respectively (Figure 7C). However, when PIMT was silencing by siRNA in cells overexpressing wild-type PIMT and cells were treated with TGF-β1, this was accompanied by a significant Slug gene inhibition, by about 38%, compared to cells overexpressing wild-type PIMT in the presence of TGF-β1 (Figure 7C). Similarly, Slug mRNA level was significantly lower, by 30%, in cells overexpressing wild-type PIMT that were also transfected with PIMT siRNA and treated with TGF-β1 compared to those transfected with control siRNA and incubated with TGF-β1 (Figure 7C). Similar patterns were observed in Slug protein expression following inhibition of wild-type PIMT by siRNA upon U-87 MG cells treatment with TGF-β1 (Figure 7E,G). Again, TGF-β1 treatments significantly induced Slug protein levels by 2.8-fold in mock cells, by 6.3-fold in cells overexpressing wild-type PIMT and by 6.8-fold in cells overexpressing wild-type PIMT and transfected with control siRNA, compared to mock cells untreated with TGF-β1 (Figure 7E,G). Interestingly, cells overexpressing wild-type PIMT then transfected by PIMT siRNA and treated with TGF-β1 exhibited a Slug protein level that was closer to that observed in mock cells treated with TGF-β1 (Figure 7E,G). Consequently, Slug protein level in cells overexpressing wild-type PIMT, transfected with PIMT siRNA, and treated with TGF-β1 was significantly reduced by 59% compared to cells overexpressing wild-type PIMT and treated with TGF-β1 and Slug protein level was lower by 62% relative to cells overexpressing wild-type PIMT and transfected with control siRNA then treated with TGF-β1 (Figure 7E,G). Together, these data on Slug gene and protein regulation supported the assumption that Slug induction by TGF-β1 in U-87 MG glioma cells was partially mediated by PIMT.

In the case of Snail expression, its mRNA level was significantly enhanced, by five-fold, in mock cells treated with TGF-β1 (Figure 7D) but the cytokine was unable to significantly induce Snail gene expression in cells overexpressing wild-type PIMT (Figure 7D). A similar observation was made in cells overexpressing wild-type PIMT and transfected with control siRNA then incubated with TGF-β1 (Figure 7D). However, adding TGF-β1 to cells overexpressing wild-type PIMT that were also transfected with PIMT siRNA led to a marked and significant six-fold induction in Snail gene expression compared to cells untreated with TGF-β1 (Figure 7D) and to a significant three-fold increase when compared to cells overexpressing wild-type PIMT and transfected with control siRNA then treated with TGF-β1 (Figure 7D). Similar significant results were revealed by Western blot analyses and densitometric quantifications when Snail protein levels were examined in cells overexpressing wild-type PIMT and in cells overexpressing wild-type PIMT and transfected with either control siRNA or PIMT siRNA compared to mock cells untreated with TGF-β1(Figure 7E,H). Together, these results confirmed that PIMT inhibitory capacity on Snail gene and protein expression dependent on TGF-β1 was reversible since it was alleviated by PIMT siRNA treatment in U-87 MG cells overexpressing wild-type PIMT. Furthermore, by reducing the amount of overexpressed wild-type PIMT by siRNA, these experiments were able to mimic previous results in U-87 MG glioma cells overexpressing mutant PIMT(D83V) by increasing both Snail mRNA and protein levels in the presence of TGF-β1.

## 3. Discussion

Glioblastomas are aggressive, vascularized, and invasive tumors. Thus, these brain tumors can be difficult to treat, and patients have a median survival of 15 months [1]. Furthermore, the blood brain barrier can filter drugs, or the GBM can develop drugs resistance. From these facts, it is clear that alternative research strategies are necessary for developing new protocols to treat these patients. EMT is well known to be activated in several cancers including in GBM [24] and molecular and cellular processes associated with EMT are essential steps in carcinogenesis. Moreover, TGF-β1 is known to induce EMT in cancer cells [25]. In particular, TGF-β1 is involved in different processes of GBM growth such as proliferation, migration, invasion, angiogenesis and suppression of immune system against tumor cells [26]. Thus, EMT pathways dependent on TGF-β1 could contain potential therapeutic targets that will be useful in GBM therapeutic arsenal. 

PIMT is an enzyme that can repair proteins with damaged aspartyl residues [4] and that has a high level in the brain [3]. We previously reported that the level of type I PIMT mRNA was stimulated by 2.3-fold in GBM tissues [12]. Furthermore, we demonstrated that PIMT protein isoform I plays a key role in processes such as adhesion, migration, and invasion of U-87 MG and U-251 MG glioma cells [10]. These findings could be critical to explore the involvement of PIMT isoform I in EMT and in improving our understanding of PIMT role in GBM growth. 

In the current study, we showed, for the first time, that PIMT was involved in EMT responses triggered by TGF-β1 treatments in U-87 MG cells, which is a suitable model for glioma [10]. Notably, when using gene array analysis, we determined that expression of Slug, Snail, and Fibronectin were upregulated while that of Twist1 was downregulated in U-87 MG cells incubated with TGF-β1. These changes in gene expression of EMT markers were supported by data obtained with Real-Time PCR assays and Western blot analysis for mRNA and protein quantification of EMT markers in U-87 MG cells treated with TGF-β1. However, TGF-β1 did not regulate PIMT gene and protein expression under conditions that regulated EMT markers in U87-MG cells. An important point of this study was that the migratory properties of U-87 cells were mediated by PIMT, since the overexpression of wild-type protein increased the migration potential while overexpression of the mutant PIMT(D83V) prevented TGF-β1 capacity to stimulate migration. However, a major discovery of this study was that PIMT isoform I upregulated both basal and TGF-β1-dependent Slug mRNA and protein expression in U-87 MG cells. Another crucial finding was that while the higher Slug mRNA and protein levels in the presence of TGF-β1 were partially mediated by PIMT isoform I, the lower Snail mRNA and protein levels when wild-type PIMT was overexpressed, suggesting that the active enzyme interfered with Snail stimulation by the cytokine. Thus, PIMT presented antagonist effects on Slug and Snail expression in response to TGF-β1 action in U-87 MG glioma cells.

Aggressive cancer cells lose their epithelial markers and instead they express mesenchymal markers. We showed that when U-87 MG glioma cells were treated with TGF-β1, they exhibited EMT markers indicating that they behave like mesenchymal cells. Cell invasion is a typical hallmark of EMT in cancer cells. Interestingly, PIMT silencing by siRNA inhibited in vitro migration of U-87 MG cells dependent on TGF-β1. In contrast, overexpressing wild-type PIMT and adding TGF-β1 had cumulative effects on migration in U-87 MG cells. On the other hand, the morphology of U-87 MG cells incubated with TGF-β1 was similar to the spindle star shape. This spindle shape was also observed in detached breast MDA-MB-231 cancer cells from ECM, and this was accompanied by a higher PIMT level [7]. Cell morphology as star shape when PIMT was downregulated by siRNA was less marked compared to control cells. However, in cells that overexpressed wild-type PIMT, the spindle shape was more pronounced compared to mock cells. Thus, the catalytic activity of PIMT seems to be involved in this EMT feature. When cancer cells acquire mesenchymal properties, their changes in cell morphology are dependent on cytoskeleton reorganization to allow migration and invasion. Previously, we demonstrated that overexpression of wild-type PIMT induced migration and invasion of U-87 MG cells [10]. Furthermore, we conducted immunofluorescence studies to show that both PIMT silencing and overexpression of wild-type PIMT led to reorganization of F-actin and microtubules [10]. On the other hand, Slug has been identified among the most overexpressed transcription factors promoting migration in human GBM when compared to normal brains by using mRNA microarray analysis [27]. In addition, overexpression of Slug enhanced migration and invasion in U-251 MG glioma cells while Slug silencing by shRNA decreased migration and invasion in U-87 MG glioma cells [27]. Our results show that Slug induction by TGF-β1 was mediated by PIMT in U-87 MG cells. Thus, in future experiments, it would be interesting to use stable U-87 MG cells that overexpressed wild-type and mutated PIMT and to inhibit Slug by siRNA and to treat these cells with TGF-β1. Then, to determine by immunofluorescence studies the effects on F-actin and microtubules polymerization. These studies will allow a better understanding of the molecular mechanisms involved in migration and invasion processes in GBM growth. 

Few studies have explored PIMT contribution to EMT, and fewer studies have analyzed whether TGF-β1 can regulate PIMT expression. Here, we found that PIMT was unaffected by either TGF-β1 and TGF-β2 at mRNA and protein levels under conditions that triggered EMT in U-87 MG cells. In contrast, in MDA-MB-231 breast cancer cells, it was demonstrated that PIMT was stimulated by a co-treatment with TGF-β1/TNF-α, which also increased mRNA levels of EMT markers including those of Snail, Slug, MMP-2, MMP-9, and Fibronectin [7]. However, multiple factors have been implicated as activators of EMT programs in addition to TGF-β1. Moreover, EMT is a multi-step process that enables cancer cells to detach from tumors and to invade distant organs. When cancer cells break away from a tumor, they need to escape from anoikis. When MDA-MB-231 cells were cultured in suspension, the mRNA levels of several EMT markers including those of Snail, Slug, MMP-2, TGF-β1 and integrin αv were upregulated [7]. PIMT mRNA level was also induced when these cells were kept in suspension [7]. Previously, we reported that PIMT amounts in U-87 MG glioma cells and in Caki-1 renal carcinoma cells were increased by cell detachment but decreased upon re-adhesion to various proteins present in the ECM [6]. Recently, we reported that overexpression of wild-type PIMT in U-87 MG cells promoted in vitro colony formation on a plastic support in the absence of ECM, suggesting that PIMT could be carcinogenic in an anchorage-independent environment as found in glioma tumors [10]. PIMT is known to act as an anti-apoptotic protein in different cell types, including in brain cells [8]. For all these reasons, it could be interesting to investigate whether the PIMT increase in U-87 MG cells when cultured in suspension would be accompanied by an EMT response by analyzing the induction of EMT markers. Furthermore, it could be relevant to determine whether PIMT would be involved in the activation of these EMT markers by silencing PIMT by siRNA and by overexpressing wild-type PIMT. Analyzing apoptotic pathways, including different caspase activities, in detached U-87 MG cells would also help to clarify the anti-apoptotic role of PIMT in anoikis in these cells during EMT. Overall, these investigations with another EMT model, where PIMT is upregulated upon U-87 MG cells detachment, would help to better understand its involvement in the molecular mechanisms in EMT pathways. 

Our results confirmed that TGF-β1 treatments in U-87 MG cells induced the expression of proteins Snail, Slug, N-Cadherin, and Fibronectin as previously reported [28,29,30]. However, in our hands, TGF-β1 treatments in U-87 MG cells inhibited the expression of Twist1. This result agrees with another study in which Twist1 was also downregulated in U-87 MG cells treated with TGF-β1 [29]. In contrast, Twist1 was induced by TGF-β1 in U-87 MG cells in a second study [30], while a third study reported that TGF-β1 treatment in these cells did not affect Twist1 level [28]. More experimental investigations will be required to clarify these contradictory data in order to improve our understanding on how Twist1 is regulated by TGF-β1 in U-87 MG glioma cells and likely its modulation by TGF-β1 in GBM growth.

A major discovery was that PIMT isoform I overexpression stimulated both basal and TGF-β1-induced Slug mRNA and protein expression in U-87 MG cells. Cells transfected with the plasmid containing wild-type PIMT significantly increased Slug gene and protein expression with about the same efficiency as a treatment with TGF-β1. On the other hand, in cells that overexpressed wild-type PIMT and were also treated with TGF-β1, Slug expression was higher than those measured in mock cells treated only with TGF-β1 and in cells that overexpressed PIMT but untreated with TGF-β1. By contrast, when the mutated form PIMT(D83V) was overexpressed, Slug mRNA and proteins levels were drastically decreased compared to cells that overexpressed wild-type PIMT in the absence of TGF-β1. Similarly, when the mutated form PIMT(D83V) was overexpressed, but cells were also treated with TGF-β1, Slug mRNA and proteins expression were significantly reduced compared to cells that overexpressed wild-type PIMT in the presence of TGF-β1. These results demonstrate the critical role of the catalytic activity of PIMT in basal and TGF-β1-dependent Slug upregulation. However, another interesting finding was that while the increase in Slug mRNA and protein expression induced by TGF-β1 was partially mediated by PIMT, this was accompanied by a decrease in Snail mRNA and protein levels when wild-type PIMT was overexpressed, suggesting that the active enzyme also interfered with Snail stimulation triggered by the cytokine. Thus, PIMT presented antagonist effects on Slug and Snail expression in response to TGF-β1 action in U-87 MG glioma cells.

The fact that overexpression of wild-type PIMT, but not that of mutated PIMT(D83V), increased basal Slug gene expression underlines that the activity of the enzyme was necessary to induce this event. Unfortunately, nothing is known on the signaling pathways regulated by PIMT that could target the expression of Slug gene. However, it is well documented that numerous post-translational modifications of an EMT transcriptional factor such as Slug could regulate its biochemical functions, including nuclear localization, protein stability, and protein-protein interactions [31]. A possible explanation of the role of PIMT is that the enzyme methylates a key protein which promotes the nuclear localization of Slug. Alternatively, PIMT may recognize specific proteins that increase Slug stability by protecting it against proteosomal degradation and thus facilitating its migration toward the nuclei. Another possibility is that a nuclear form of PIMT acts on proteins that are already bound to Slug gene promoter and thus PIMT regulates Slug gene expression through them. Thus, chromatin immunoprecipitation (ChIP) assays should be conducted to validate PIMT binding to Slug gene promoter. All these experimental possibilities to analyze how PIMT regulates basal Slug gene expression could provide critical insights, since Slug mRNA expression has been correlated with glioma tumor grade, and Slug overexpression increased migration and invasion in U-251 MG and U-87 MG human glioma cell lines [27].

PIMT protein levels were unaffected when U-87 MG cells were treated with TGF-β1 and TGF-β2. Thus, to understand PIMT functions in U-87 MG cells in the presence of TGF-β1, we need to explore which signaling pathways the cytokine uses to regulate PIMT activity rather than identify which mechanisms are enhancing its amount. TGF-β1 could induce canonical SMAD and noncanonical signaling pathways to regulate EMT [32]. Among these latter signaling pathways, TGF-β1 can activate the PI3K and the Akt signaling pathway or to induce ERK1/2 pathway through Src kinase and RAS. Recently, a new method to screen kinases and interacting substrates at a large scale has shown that kinases Lyn and Fyn phosphorylated PIMT on tyrosine 195 and tyrosine 213 [33]. Lyn and Fyn are two members of Src-family kinase and their signaling impacts on multiple tumor properties have been studied, notably in the context of GBM. For instance, phosphorylation analysis has established Lyn kinase activity to be significantly elevated in GBM [34]. Similarly, Fyn signaling pathways that regulate EMT also contribute to cancer progression, including in glioma growth [35]. Thus, a possible scenario explaining the mode of action of PIMT to mediate the effects of TGF-β1 is that the cytokine activates Lyn or Fyn kinases in U-87 MG cells and one of these kinases could phosphorylate PIMT on tyrosine 195 and/or tyrosine 213. Phosphorylated PIMT could methylate a protein which would interact with and promote the nuclear localization of Slug. Thus, immunoprecipitation experiments should be done to determine whether the level of phosphotyrosine PIMT is increased upon TGF-β1 treatment in U-87 MG cells.

While Slug gene and protein expression were induced by TGF-β1, and these events were partly mediated by PIMT, there was in parallel a reduction in Snail gene and protein levels under these conditions. Thus, PIMT overexpression exhibited antagonist effects on Slug and Snail expression in response to TGF-β1 action in U-87 MG glioma cells. In a broad sense, for Slug upregulation by TGF-β1, PIMT acts through activation mechanisms while for Snail downregulation by TGF-β1, PIMT behaves through inhibitory mechanisms. From these antagonist behaviors, possible explanations for Snail inhibition include opposite mechanisms to those previously suggested above to be involved in Slug upregulation.

Despite our findings on the potential role of PIMT isoform I in growth of human GBM, our study has some limitations. Notably, GBM are characterized by a marked heterogeneity at the cellular and molecular levels [36]. Thus, the results found in U-87 MG cells treated with TGF-β1 should be reproduced in other glioma cell lines with different aggressiveness and phenotypes to support the oncogenic features of PIMT isoform I in glioma cells. In addition, experiments that focus on PIMT functions in vivo should be done to demonstrate its biological relevance. For example, an orthotopic nude mouse model of GBM with xenografts of human GBM cell lines that overexpresses wild-type and mutated PIMT will help to better determine the prognostic impact of the enzyme in GBM.

## 4. Materials and Methods

### 4.1. Antibodies

The rabbit monoclonal anti-PIMT antibody (1:5000) was purchased from Origene (Rockville, MD, USA). Recombinant human TGF-β1 was purchased from ThermoFisher Scientific (Waltham, MA, USA). Rabbit antibodies against Snail, Slug and N-cadherin (1:1000) and the mouse antibody against GAPDH (glyceraldehyde 3-phosphate dehydrogenase) (1:25,000) were purchased from Cell signaling Technology (Beverly, MA, USA). The monoclonal antibody against Twist1 (clone number: Twist2C1a) (1:200) was from Santa Cruz Biotechnologies (Santa Cruz, CA, USA). The mouse antibody against Fibronectin (1:1000) was from BD Biosciences (Mississauga, ON, Canada). The donkey anti-rabbit and goat anti-mouse peroxidase-conjugated antibodies (1:2500) were purchased from Jackson Immunoresearch Laboratories (West Grove, PA, USA). 

### 4.2. Cell Culture 

A human glioma U-87 MG cell line was obtained from American Type culture Collection (Manassas, VA, USA). Cells were cultured in a humidified atmosphere containing 5% CO_2_ at 37 °C. Cells were grown in Eagle’s Minimum Essential Medium (Wisent, St-Bruno, QC, Canada) completed with 1 mM sodium pyruvate and 10% fetal bovine serum (Wisent, St-Bruno, QC, Canada). U87-MG cells were treated with 10 ng/mL of TGF-β1 during 24 h in free medium. 

### 4.3. PCMT1 Gene Transfections 

The human *PCMT1* gene tagged with the Myc/DDK in N-terminal and contained in PCMV6 entry plasmid was purchased from Origene (Rockville, MD, USA). A PCR method was used to delete the N-terminal Myc/DDK tag of *PCMT1* gene [8]. This untagged wild-type *PCMT1* gene served to construct a mutated inactive form of PIMT, PIMT(D83V), by point mutations by two-step PCR directed mutagenesis as described in [8]. The plasmids were extracted from E. coli bacteria using mini Prep kit from Qiagen conforming to the manufacturer’s instructions (Valencia, CA, USA). To overexpress PIMT in U-87 MG cells, 100,000 cells were seeded into 6-well plates. After 48 h, cells were transfected with 1 µg of the empty pCMV6 vector, the wild-type pCMV6-PCMT1 plasmid, or the inactive mutant pCMV6-PCMT1(D83V) plasmid using TurboFectine 8.0 reagent, from OriGene, (Rockville, MD, USA) to 24 h at 37 °C. New medium containing 400 μg/mL G418 antibiotic (Wisent, St-Bruno, QC, Canada) was added to select cells that integrated plasmids. To maintain stable cells lines that overexpress wild-type and mutated PIMT plasmids, 200 μg/mL G418 antibiotic was added to them. 

### 4.4. Identification of U-87 MG Cells Colonies Overexpressing Wild-Type PIMT

To identify cell colonies overexpressing wild-type PIMT at the highest levels, about 500 U-87 MG cells transfected with pCMV6-PCMT1 plasmid were individualized and cultured on a dish of 100 mm for 10 days at 37 °C and 5% CO_2_. Complete culture medium was changed every 3 days. After colony formation, cells are washed with phosphate-buffered saline (PBS). Sterile clone cylinders (Sigma-Aldrich, Oakville, ON, Canada) were used to extract each colony with trypsin. U-87 MG cells were resuspended in a 96-well microplate until 80% confluence. Then, cells were resuspended on a 24-well microplate following by an amplification on a 6-well microplate. Finally, U-87 MG cells overexpressing wild-type PIMT were cultured in F25 flasks. After colony formation, cells were always cultured with 200 μg/mL G418 antibiotic. PIMT levels were detected by Western blots to identify U-87 MG cells with higher overexpression. 

### 4.5. Transfection Method by RNA Interference

PIMT siRNA and AllStars negative control siRNA were purchased from Qiagen (Valencia, CA, USA). U-87 MG cells at a density 100,000 were seeded into 6 wells plates and grown to 50% confluence. A mixture of 40 nM of PIMT siRNA or the negative control and Lipofectamine 2000 were added to cells for 72 h then a replacement of new medium with 10% of serum after 24 h according to manufacturer’s instruction (Burlington, ON, Canada). Similarly, to inhibit the overexpression of wild-type pCMV6-PCMT1 in stable cell lines, we used the same conditions for PIMT siRNA and control siRNA treatments as described above for U87-MG cells transfected with siRNA.

### 4.6. Soluble Protein Extraction from Cells

A density of 100,000 U-87 MG cells was seeded into wells of 6-well tissue culture dishes, cells transfected with PIMT siRNA for 72 h or 80% confluence of the stables cells lines (the wild-type pCMV6-PCMT1 plasmid, the inactive mutant pCMV6-PCMT1(D83V) plasmid and pCMV6 vector). Ice-cold PBS was added to cells treated as described before and cells were scraped in ice-cold RIPA buffer (50 mM Tris pH 7.50, 150 mM NaCl, 0.1% SDS, 1% Nonidet P-40, 0.5% Deoxycholate) with a protease inhibitor cocktail (Calbiochem, San Diego, CA, USA) and phosphatase inhibitors with 1 mM sodium fluoride (NaF) and 1 mM sodium orthovanadate (Na_3_VO_4_). Cell lysates were kept on ice for 30 min and soluble proteins were recovered from lysates by centrifugation at 10,000× *g* for 10 min at 4 °C. The concentration of proteins was quantified by using bovine serum albumin as a standard and the Pierce micro-bicinchoninic acid (micro BCA) Assay Kit (Pierce, Rockford, IL, USA). 

### 4.7. Human Epithelial to Mesenchymal Transition Signaling Pathways PCR Array Analysis

The human epithelial to mesenchymal transition (EMT) signaling pathways PAHS-090ZD-6 RT^2^ Profiler PCR Arrays was purchased from Qiagen (Valencia, CA, USA) and was used according to the manufacturer’s protocol. The cDNA from U-87 MG cells treated or untreated with TGF-β1 was prepared by RT-PCR, mixed with SooFast EvaGreen Supermix (Bio-rad, Hercules, CA, USA), and measured by CFX Connect Real-Time System (Bio-Rad, Hercules, CA, USA) to obtain the CT values of genes. The SsoFast EvaGreen Supermix bound to DNA and the fluorescence was correlated to each cycle of amplifications. Relative gene expressions were evaluated by 2 ^−ΔΔCT^ method (= 2^−(experimental condition ΔCT-control condition ΔCT)^) that calculated the normalized ratio and CT (cycle threshold) defined as the number of cycles necessary for the fluorescent signal to cross the detection threshold. The normalized ΔCT, was obtained by the number of cycles between the CT of interest gene and CT of the control gene, GAPDH. Results of CT values were analyzed using the PCR Array Data Analysis Template from Qiagen (http://geneglobe.qiagen.com/us/analyze/, accessed on 13 November 2019). 

### 4.8. Quantitative PCR Analysis 

A density of 100,000 U-87 MG cells was plated into wells of 6-well plates with either the stables cells lines (the wild-type pCMV6-PCMT1 plasmid, the inactive mutant pCMV6-PCMT1(D83V) plasmid and pCMV6 vector) grown for 80% confluence or with cells transfected with PIMT siRNA constructions as described above. These cells were treated with 10 ng/mL of TGFβ-1 in serum-free medium and incubated at 37 °C and 5% CO_2_ for 24 h. Total RNA was extracted using TRIzol reagent (Life Technologies, Gaithersburg, MD, USA). A high-capacity reverse transcription kit (Applied Biosystems, Foster City, CA, USA) was used to synthetize 2 µg of complementary DNA from total RNA according to the manufacturer’s protocol. The amplifications were produced by Taq polymerase and quantified using SsoFast EvaGreen Supermix that bound the double stranded DNA (Bio-Rad, Hercules, CA, USA) and measured by CFX Connect Real-Time System (Bio-Rad). The QuantiTect primers was purchased from Qiagen (Valencia, CA, USA): PCMT1 (Hs_PCMT1_1_SGQT00034797), Slug (Hs_SNAI2_1_SGQT00044128), Snail (Hs_SNAI1_1_SGQT00010010), Twist (HS_TWIST1_1_SGQT00011956), N-cadherin (HS_CDH2_1_SGQT00063196), Fibronectin (HS_FN1_1_SGQT00038024), GAPDH (HS_GAPDH_1_SGQT00079247) and PPIA (HS_PPIA_1_SGQT00052311). The GAPDH and PPIA were used as controls. Reactions were done in triplicate in each experiment. Fluorescence was quantified into each cycle (C_T_) for exponential amplification using CFX Manager Software version 2.1 from Biorad and 2 ^−ΔCT^ method that expressed the relative quantified value (RQV), where the ΔC_T_ value was calculated by the difference of CT values between gene of interest and control gene.

### 4.9. Western Blotting

An amount of 15 µg proteins mixed in Laemmli buffer was separated by two different gel types: 12.5% SDS PAGE gels or gradient gels of 4–20% Mini-PROTEAN TGX ^TM^ to detect N-cadherin, and Fibronectin. Proteins were transferred to 0.45 µM pore diameter polyvinylidene difluoride (PVDF) membranes (Pall Life Sciences, Pensacola, FL, USA). The non-specific sites in the membrane were blocked for 1 h in TBS (20 mM Tris-HCl pH 7.5, 150 mM NaCl) containing 0.1% (*v*/*v*) Tween 20 (TBS-T) and 5% (*w*/*v*) milk powder. Then, the membranes were incubated with different primaries antibodies overnight at 4 °C. The membranes were incubated for 1 h at room temperature with the secondary antibodies (1:2500), horseradish-peroxidase-coupled donkey anti-rabbit IgG or goat anti-mouse IgG in TBS-T containing 5% milk powder. The proteins from PVDF membranes were revealed using HyGlo Chemiluminescent HRP Antibody Detection Reagent (Denville Scientific, Metuchen, NJ, USA) and exposed to HyBlot CL Autoradiography films (Denville Scientific, Metuchen, NJ, USA). The autoradiograms were scanned to determine proteins levels by densitometric analysis using Image J Software (NIH, Bethesda, MD, USA).

### 4.10. Cell Migration Assays

Wound healing assays were performed in 6-well plates. A total of 100,000 U-87 MG cells were allowed to reach 100% confluence then a scratch was performed using a 200 μL sterile pipette tip on cell monolayers. Cells were washed with culture medium to remove floating cells after a scratch. To allow migration, cells were treated with 10 ng/mL of TGFβ-1 in serum-free medium and incubated at 37 °C for 24 h. Cells were washed with cold PBS and fixed with ice-cold 100% (*v*/*v*) methanol. At t = 0 cells were unstained while at t = 24 h they were stained with 0.5% crystal violet in 25% (*w*/*v*) methanol. Migration areas were photographed at time 0 h and 24 h using camera Retiga 1300 and the Nikon microscope model TE2000-U. The free migration areas were quantified using Image J Software (NIH, Bethesda, MD, USA).

### 4.11. Effect of TGFβ-1 on Cell Morphology 

A total of 100,000 U-87 MG cells transfected with PIMT siRNA or control siRNA and stable cells overexpressing wild-type PIMT or mutated PIMT(D83V) were treated, or not, with 10 ng/mL TGFβ-1 in serum-free medium for 24 h at 37 °C. The cells were photographed using camera Retiga 1300 and the Nikon microscope model TE2000-U to analyze changes in cell morphology induced by TGFβ-1.

### 4.12. Statistical Data Analysis

Image J software was used to analyze densitometric of immunodetected proteins and cellular assays. Results are representative of at least three independent experiments for means results ± SD. For two groups, the statistical analyses were validated by Student’s unpaired t test. For more than two groups, the One Way Anova followed by the Benferroni test are used. 

The statistical analyses of all experiences were confirmed using GraphPad Prism 5 (GraphPad Software, San Diego, CA, USA). 

## 5. Conclusions

In summary, this study supports a possible oncogenic role for PIMT isoform 1 since we showed that it was promoting the migration of U-87 MG glioma cells during an EMT-like process triggered by TGF-β1. Furthermore, our investigations revealed that PIMT overexpression during the EMT response in U-87 MG cells led to an antagonist regulation of transcriptional factors Slug and Snail at mRNA and protein levels upon TGF-β1 treatments. However, further studies are needed to identify which signaling pathways stimulated by TGF-β1 act on PIMT, and which substrates are methylated by PIMT, to better understand how PIMT could upregulate Slug and inhibit Snail expression. The present findings suggest that targeting PIMT action may be a novel therapeutic method for a better GBM therapy.

## Data Availability

The datasets used and/or analyzed during the current study are available from the corresponding author and Supplementary Materials on reasonable request.

## Supplementary Materials

The following supporting information can be downloaded at: https://www.mdpi.com/article/10.3390/ijms23105698/s1.

## Abbreviations

BSA: bovine serum albumin; ChIP, chromatin immunoprecipitation; ECM, extracellular matrix; EMT, epithelial mesenchymal transition; ER, estrogen receptor; GBM, Glioblastoma; GMT, glial to mesenchymal transition; HUVECs, human umbilical vein endothelial cells; MMPs, matrix metalloproteinases; PBS, phosphate-buffered saline; PIMT, Protein L-isoaspartyl (D-aspartyl) methyltransferase; PVDF, polyvinylidene difluoride; qRT-PCR, quantitative reverse-transcription polymerase chain reaction; SDS-PAGE, sodium dodecyl sulfate polyacrylamide gel electrophoresis; TBS-T, Tris-buffered saline containing 0.1% Tween 20; VEGF, vascular endothelial growth factor.
