# Supplementary figures and images for: The Protein L-Isoaspartyl (D-Aspartyl) Methyltransferase Regulates Glial-to-Mesenchymal Transition and Migration Induced by TGF-β1 in Human U-87 MG Glioma Cells
Source: Int J Mol Sci. 2022 May 19;23(10):5698. doi: 10.3390/ijms23105698 (PMC9146343; doi:10.3390/ijms23105698)

Figure S1-Supplementary

Control

TGF-β1

n1

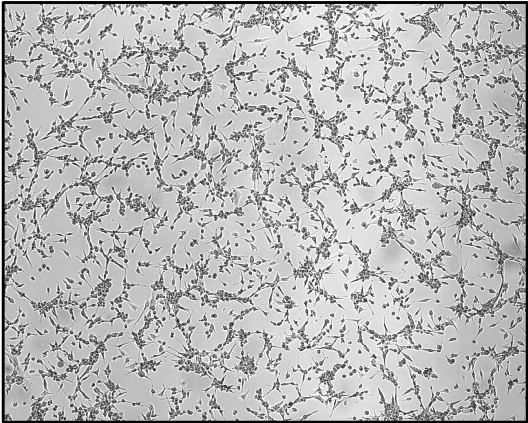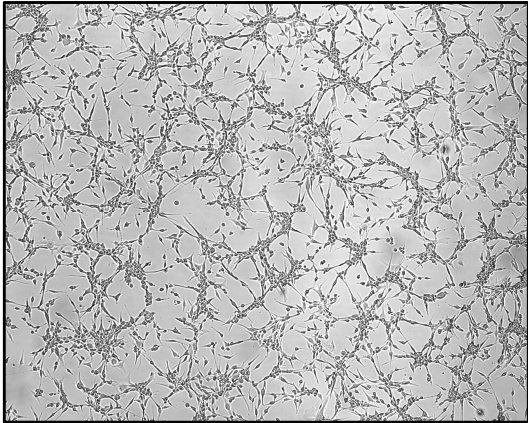

n2

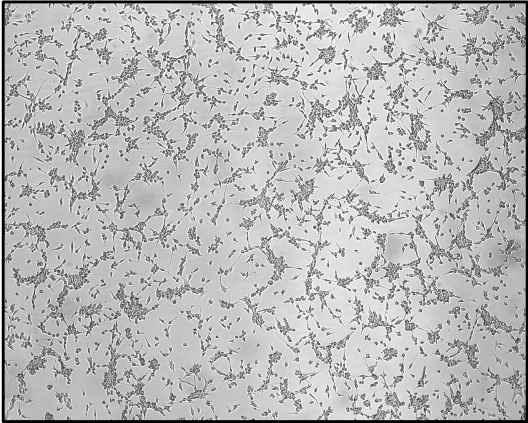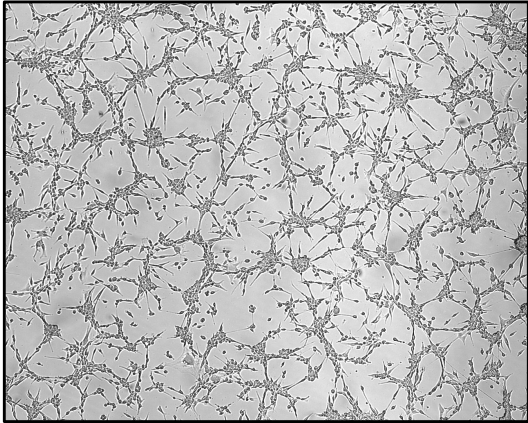

Figure S1-Supplementary

Control

TGF- $\beta$ 1

n3

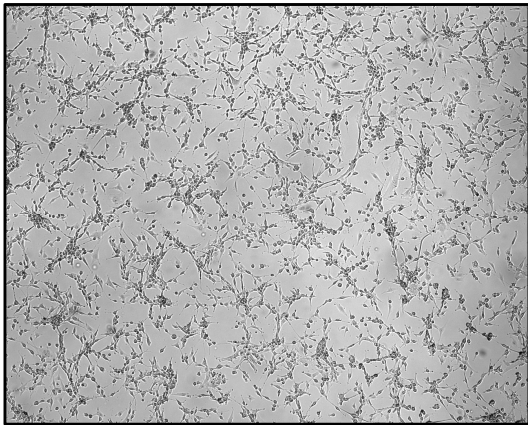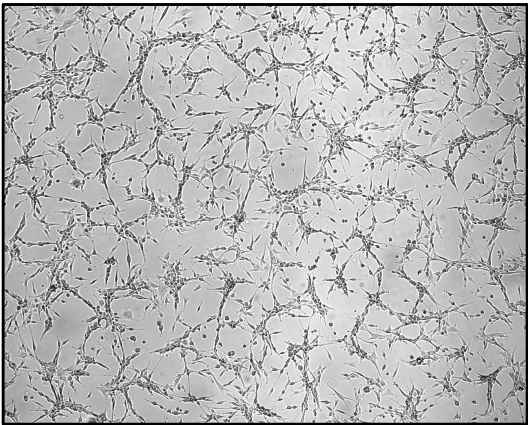

n4

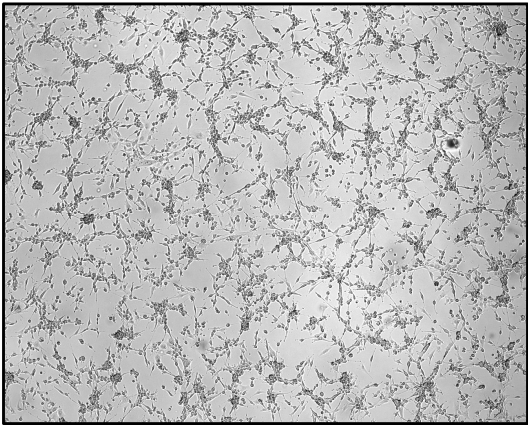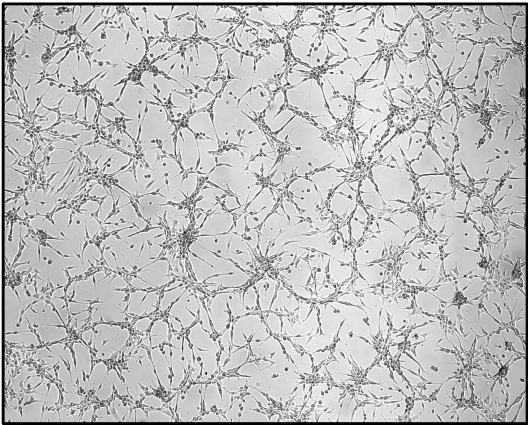

Supplement: Supplementary file 1 [file ijms-23-05698-s001.zip › Figure S1 supplementary data.pdf]
